# Identification and Functional Analysis of Key microRNAs in the Early Extrauterine Environmental Adaptation of Piglets

**DOI:** 10.3390/ijms26031316

**Published:** 2025-02-04

**Authors:** Mingxing Wen, Jing Li, Wanling Qiu, Jinwei Zhang, Keren Long, Lu Lu, Long Jin, Jing Sun, Liangpeng Ge, Xuewei Li, Mingzhou Li, Jideng Ma

**Affiliations:** 1 State Key Laboratory of Swine and Poultry Breeding Industry, College of Animal Science and Technology, Sichuan Agricultural University, Chengdu 611130, China; wenmx0126@163.com (M.W.); lijing09002022@163.com (J.L.); qiuwanling2016@163.com (W.Q.); keren.long@sicau.edu.cn (K.L.); lu.lu@sicau.edu.cn (L.L.); longjin@sicau.edu.cn (L.J.); xuewei.li@sicau.edu.cn (X.L.); mingzhou.li@sicau.edu.cn (M.L.); 2 Chongqing Academy of Animal Sciences, Chongqing 402460, China; jinweizhang50@163.com (J.Z.); sunjing85026@163.com (J.S.); geliangpeng1982@163.com (L.G.); 3 Key Laboratory of Pig Industry Sciences, Ministry of Agriculture, Chongqing 402460, China; 4 Chongqing Key Laboratory of Pig Industry Sciences, Chongqing 402460, China

**Keywords:** piglets, early postnatal adaptation, microRNA, gluconeogenesis

## Abstract

Neonatal mammals must rapidly adapt to significant physiological changes during the transition from the intrauterine to extrauterine environments. This adaptation, particularly in the metabolic and respiratory systems, is essential for survival. MicroRNAs (miRNAs) are small noncoding RNAs that regulate various physiological and pathological processes by binding to the 3′ untranslated regions of mRNAs. This study aimed to identify miRNAs involved in the early extrauterine adaptation of neonatal piglets and explore their functions. We performed small RNA sequencing on six tissues (heart, liver, spleen, lung, multifidus muscle, and duodenum) from piglets 24 h before birth (day 113 of gestation) and 6 h after birth. A total of 971 miRNA precursors and 1511 mature miRNAs were identified. Tissue-specific expression analysis revealed 881 tissue-specific miRNAs and 164 differentially expressed miRNAs (DE miRNAs) across the tissues. Functional enrichment analysis showed that these DE miRNAs are significantly enriched in pathways related to early extrauterine adaptation, such as the NFκB, PI3K/AKT, and Hippo pathways. Specifically, miR-22-3p was significantly upregulated in the liver post-birth and may regulate the PI3K/AKT pathway by targeting AKT3, promoting gluconeogenesis, and maintaining glucose homeostasis. Dual-luciferase reporter assays and HepG2 cell experiments confirmed AKT3 as a target of miR-22-3p, which activates the AKT/FoxO1 pathway, enhancing gluconeogenesis and glucose production. Furthermore, changes in blood glucose and liver glycogen levels in newborn piglets further support the role of miR-22-3p in glucose homeostasis. This study highlights the importance of miRNAs, particularly miR-22-3p, in the early extrauterine adaptation of neonatal piglets, offering new insights into the physiological adaptation of neonatal mammals.

## 1. Introduction

Neonatal mammals must rapidly adapt to drastic changes from the intrauterine environment to the extrauterine environment at birth [1]. This process poses new challenges to the physiological functions and metabolic activities of the organism [2,3,4,5]. For example, the metabolic system of newborns must adapt to the sudden cessation of placental nutrient supply, maintaining energy balance by activating mechanisms such as gluconeogenesis [6,7]. The respiratory system must transition from relying on maternal oxygen to independent breathing, with the expansion of alveoli and the decrease in pulmonary vascular resistance being key changes in this process [3,8]. Although some progress has been made in the study of neonatal metabolic and respiratory system adaptability, research on the role of miRNAs in regulating these physiological changes is relatively limited.

miRNAs are a class of endogenous noncoding small RNA molecules [9] that regulate gene expression by binding to the 3′ untranslated region (3′ UTR) of mRNAs [10,11,12]. miRNAs play essential roles in regulating physiological processes such as cell differentiation, proliferation, and metabolism [13,14,15,16]. In the metabolic and respiratory systems of newborns, specific miRNAs participate in the regulation of energy metabolic pathways and the establishment of respiratory function by targeting key metabolic enzymes and transcription factors. For example, miRNA-22-3p has been found to play a role in hepatic gluconeogenesis, affecting the PI3K/AKT signalling pathway by targeting the AKT3 gene [17]. Moreover, miRNAs have been shown to regulate energy metabolism, including lipid metabolism and insulin sensitivity, by modulating specific metabolic enzymes and transcription factors during the postnatal transition [18,19,20]. Similarly, miRNAs are involved in regulating respiratory function by influencing pulmonary development and the expansion of alveolar structures [21,22]. Despite these insights, comprehensive studies on the regulatory roles of miRNAs in the early extrauterine environmental adaptation of newborns, particularly in piglets, remain limited. Further research is needed to fully understand their impact on neonatal physiological adaptation and health.

This study used piglets as a model and small RNA sequencing technology to screen and identify miRNAs associated with the early extrauterine environmental adaptation of newborn piglets and further validated the functions of these miRNAs through in vitro experiments. We conducted an in-depth analysis of six key tissues, including the heart, liver, spleen, lungs, longissimus dorsi muscle, and duodenum, from piglets 24 h before birth and 6 h after birth. This study aimed to elucidate the regulatory mechanisms of miRNAs in the early extrauterine adaptation of newborn piglets, offering new insights into mammalian postnatal physiological adaptation and a theoretical foundation for improving early feeding management and health protection in piglet production.

In this study, we identify a series of miRNAs related to the extrauterine adaptation of newborn piglets and explore their specific roles in regulating gluconeogenesis, energy metabolism, and organ development. These findings not only enrich our understanding of miRNA functions but also may provide potential molecular markers and intervention targets for early feeding management and health protection of piglets. Furthermore, the findings of this study may provide important references for research on the early growth and development and environmental adaptability of other mammals.

## 2. Results

### 2.1. Data Summary

To explore the role of miRNAs in the adaptive mechanisms of different tissues and organs in piglets to the early extrauterine environment, we selected female piglets 24 h before birth (i.e., 113 days of gestation, the day before the expected delivery date) and female piglets 6 h after birth (before ingesting colostrum), with three individuals per group. Six types of tissues were collected from each individual (heart, liver, spleen, lungs, longest back muscle, and duodenum). Due to the total RNA quality of one postnatal spleen sample not meeting the standard, a total of thirty-five samples were ultimately subjected to small RNA sequencing library construction. This process generated a total of 491.16 million (M) raw reads, with an average of 14.03 M per sample (standard deviation of 1.38 M). After rigorous data cleaning, we obtained 467.33 M clean reads for miRNA identification, with an average of 13.35 M per sample (standard deviation of 1.6 M). The length distribution of the clean reads peaked at 22 nt (accounting for 37.63%) and was distributed mainly between 21 and 24 nt (accounting for 76.25%) (Supplementary Figure S1). These clean reads were aligned with the pig reference genome, and the results revealed a similar number of aligned reads in pre- and postnatal samples (Supplementary Figure S2), ensuring the consistency of subsequent analyses.

Through a meticulous data screening process, we identified a total of 971 precursor miRNAs (pre-miRNAs) and 1511 mature miRNAs. Some precursor miRNAs only produce mature sequences from the 5′ end or the 3′ end, whereas others can process both the 5′ end (miRNA-5p) and the 3′ end (miRNA-3p) of mature miRNAs simultaneously. After aligning these mature miRNAs with the annotated mature miRNA sequences of pigs and other mammals in the miRBase database, we found that 444 miRNAs were completely identical to the annotated sequences of pigs and were classified as “Known” miRNAs; 697 miRNAs had seed sequences that were identical to the annotated mature miRNAs of other mammals and were categorized as “Conserved” miRNAs; and 370 miRNAs did not match any annotated sequences and were classified as “Novel” miRNAs (Table 1).

To explore the genomic origins of miRNAs, we conducted a comparative analysis of miRNA precursor sequences with various genomic elements in the pig genome. Among the 971 precursor miRNAs, 516 precursor miRNAs (53.14%) were located in intronic regions, 319 precursor miRNAs (32.85%) were located in intergenic regions, and only 1 precursor miRNA (0.1%) was found in an exon region. Considering the alternative splicing characteristics of protein-coding genes, 135 precursor miRNAs (13.9%) were found to be located simultaneously in both introns and exons of the same gene (Supplementary Figure S3).

### 2.2. miRNA Expression Profiling

**Figure 1:**
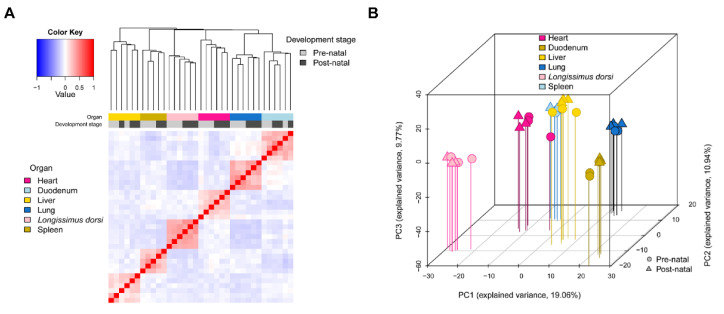
Analysis of miRNA expression profiles. (**A**) Pearson correlation matrix of the miRNA expression profiles. (**B**) Principal component analysis (PCA) of the miRNA expression profiles. The proportion of variance explained by the principal components (PCs) is indicated in parentheses; vertical lines of different colours map the plotted points onto the x/y plane, representing the dispersion of the points based on the first and second principal components.

To study the expression patterns of miRNAs in tissues before and after birth in piglets, we calculated the Pearson correlation coefficients among samples and performed principal component analysis. The results of the Pearson correlation coefficient matrix are shown in Figure 1A. The samples were first clustered according to tissue type, and then within each tissue category, the samples were further clustered based on the developmental time points before and after birth, indicating that the transition from the intrauterine to the extrauterine environment had a clear effect on miRNA expression, leading to distinct expression patterns before and after birth. The close clustering of biological replicates demonstrated the high reliability of miRNA sequencing in this study.

The results of the principal component analysis (Figure 1B) revealed that the first principal component (PC1) accounted for 19.06% of the variance, the second principal component (PC2) accounted for 10.94%, and the third principal component (PC3) accounted for 9.77% of the variance. Together, these components explain 39.77% of the variance, providing a good interpretation of the data. The different tissue categories showed clear differentiation of PC1, and samples from different developmental stages also exhibited good differentiation of PC2 and PC3. These results are consistent with the results of the Pearson correlation coefficient matrix, indicating that the differences between tissues are significantly greater than the differences between developmental stages at the transcriptome level.

### 2.3. Tissue-Specific Analysis

**Figure 2:**
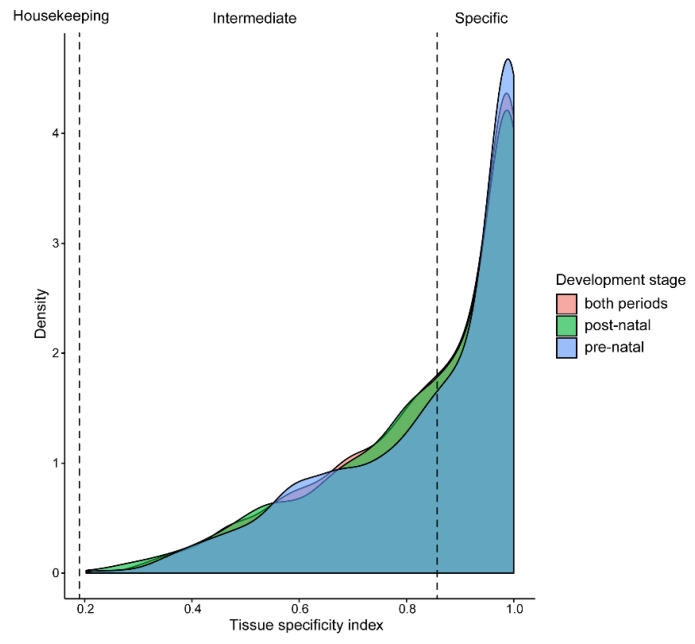
Distribution curve for TSI of miRNAs. The vertical dotted lines correspond to the threshold originally proposed for defining housekeeping and specifically expressed transcripts with TSI values < 0.15 and > 0.85, respectively.

To conduct an in-depth analysis of the tissue-specific expression patterns of miRNAs before and after birth in piglets, we used two quantitative metrics, the tissue-specific index (TSI) and the tissue-specific score (TSS), to evaluate the tissue specificity of miRNAs and their expression differences across various tissues. To assess the potential impact of two developmental time points—before and after birth—on tissue specificity, we calculated three sets of TSI values for each miRNA on the basis of the expression data of 18 prenatal samples, 17 postnatal samples, and the overall expression data covering 35 samples (Figure 2). The results revealed that the TSI distribution curves of the three groups were highly consistent, indicating that the developmental stage had a limited impact on the tissue-specific expression of miRNAs. Through a comprehensive analysis of the 35 samples, we found that 58.31% (881/1511) of the miRNAs exhibited tissue-specific expression, and 630 miRNAs presented non-specific expression patterns, indicating that they did not belong to either tissue-specific or housekeeping types. No miRNAs with housekeeping gene characteristics were found in this study.

**Figure 3:**
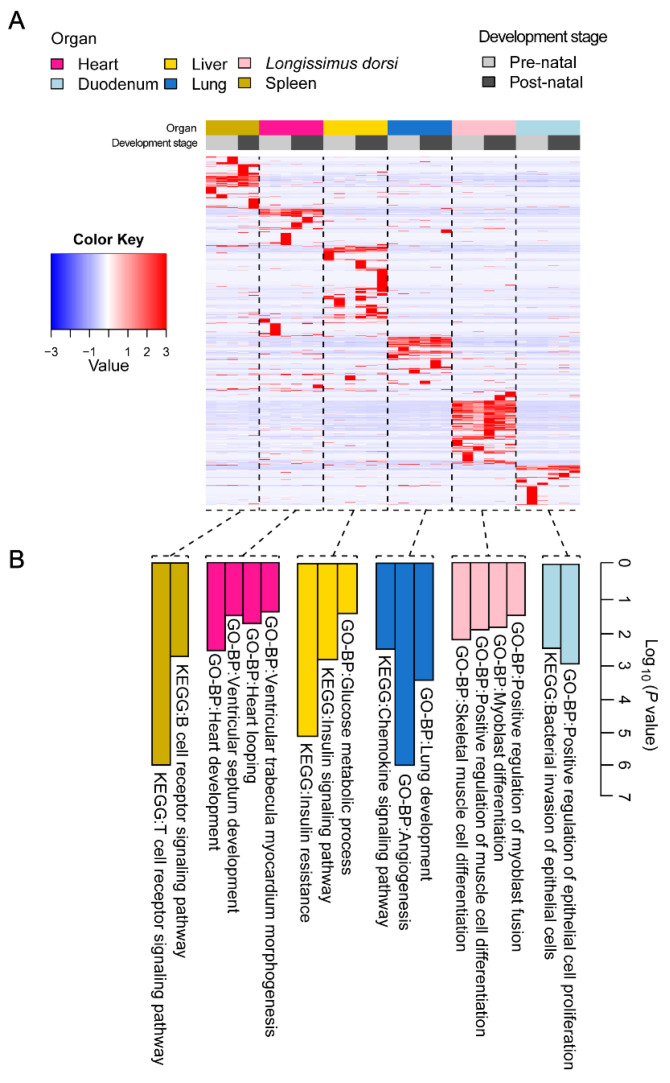
Functional pattern of tissue specificity for miRNAs. (**A**) Hierarchical clustering of miRNAs. (**B**) Functional enrichment of tissue-specific expression clusters.

To elucidate the functions of tissue-specific miRNAs in different tissues, we first performed an expression clustering analysis on these miRNAs (Figure 3A) and found that they had relatively high expression levels in their specifically expressed tissues and could form clusters on the basis of expression specificity. We subsequently used TargetScan to predict the target genes of each tissue-specific miRNA and conducted functional enrichment analysis on these target genes using DAVID software (V.2023q3) (Figure 3B). The analysis revealed that these target genes were significantly enriched in tissue-specific signalling pathways and GO terms. For example, the target genes of heart-specific miRNAs were significantly enriched in pathways related to heart development (135 genes, *p* = 2.08 × 10^−3^) and development of the cardiac ventricular septum (24 genes, *p* = 2.42 × 10^−2^). The target genes of the longest back-muscle-specific miRNAs were significantly enriched in processes such as skeletal muscle cell differentiation. The target genes of spleen-specific miRNAs were significantly enriched in immune-related pathways such as the T-cell receptor signalling pathway. The target genes of the lung-specific miRNAs were significantly enriched in respiratory system-related pathways, such as the chemokine signalling pathway. The target genes of duodenum-specific miRNAs were significantly enriched in biological processes related to epithelial cells. The target genes of liver-specific miRNAs were significantly enriched in metabolism-related pathways such as insulin resistance. These findings suggest that tissue-specific miRNAs may play a key role in maintaining the basic functions of organs by regulating the expression of protein-coding genes.

### 2.4. Differential Expression Analysis of miRNAs

To understand how newborn piglets adapt to the early extrauterine environment through the transcriptional regulation of miRNAs, we compared the miRNA transcription levels in six different tissues between pre- and postnatal piglets. We identified a total of 223 differentially expressed miRNAs (Table 2, Supplementary Table S1) across these tissues. Among them, the duodenum had the greatest number of differentially expressed miRNAs (60), followed by the lung (38), liver (37), heart (34), spleen (33), and longest back muscle (21). These findings highlight the significant role of intestinal miRNAs in the postnatal adaptation process of newborn piglets.

**Figure 4:**
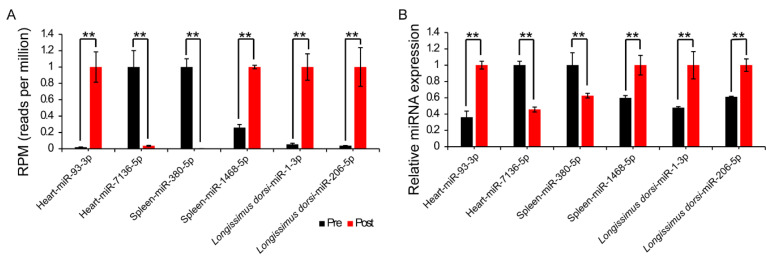
qRT-PCR validations of DE miRNA expression patterns in different tissues. (**A**) DE miRNA expression patterns detected by RNA-seq. (**B**) DE miRNA expression patterns detected by qRT-PCR. Significance levels: ** *p* < 0.01.

To verify the accuracy of the data obtained from the small RNA-seq sequencing, we randomly selected six differentially expressed miRNAs in the heart, spleen, and longest back muscle samples and independently validated them through qRT–PCR. The results shown in Figure 4 demonstrate that the validation results of qRT–PCR are highly consistent with the expression trends of the small RNA-seq data.

Newborns face the significant challenge of birth and must rapidly adapt to extrauterine life. To explore whether these changes lead to differentially expressed miRNAs showing similar expression patterns across different tissues, we conducted tissue-specific analysis on these miRNAs. The analysis revealed that the proportion of differentially expressed tissue-specific miRNAs before and after birth was 71.52%, which was significantly greater than the 56.72% for nondifferentially expressed miRNAs (Supplementary Figure S4). These findings suggest that miRNAs with tissue specificity that are differentially expressed before and after birth may play a key role in promoting the unique postnatal adaptiveness of each tissue. Detailed information on the tissue-specific differentially expressed miRNAs identified in each tissue is listed in Supplementary Table S2.

When analyzing the conservation of miRNA expression patterns before and after birth across different tissues in piglets, we found that the expression patterns of most differentially expressed miRNAs (DE miRNAs) were not conserved across tissues; specifically, 119 DE miRNAs (accounting for 72.56%) were differentially expressed in only one tissue before and after birth (Table 3). In addition, 33 miRNAs (20.12%) were differentially expressed in two tissues, 10 (6.10%) in three tissues, and only 2 (1.22%) in four tissues. No miRNAs were observed to be differentially expressed simultaneously in all five or six tissues. Notably, tissue-specific miRNAs were significantly enriched among DE miRNAs with nonconserved differential expression patterns before and after birth across tissues (chi-square test, *p* = 8.69 × 10^−4^; Supplementary Figure S5).

To gain a deeper understanding of the functions of the 94 tissue-specific and nonconserved differentially expressed miRNAs (DE miRNAs) across various tissues, we first predicted the target genes of these DE miRNAs in each tissue and then performed a functional enrichment analysis of the predicted target genes (Supplementary Table S3). The results of the functional enrichment analysis revealed that these DE miRNAs are involved primarily in regulating tissue-specific biological processes within their respective tissues. For example, in cardiac tissue, the target genes were significantly enriched in pathways related to heart development (127 genes, *p* = 9.43 × 10^−4^), cardiac circulation (47 genes, *p* = 2.69 × 10^−3^), development of the cardiac ventricular septum (22 genes, *p* = 4.29 × 10^−2^), the adrenergic signalling pathway (95 genes, *p* = 1.08 × 10^−2^), and the fibroblast growth factor receptor signalling pathway (57 genes, *p* = 2.86 × 10^−2^); in the liver, the target genes were significantly enriched in insulin resistance (90 genes, *p* = 3.58 × 10^−7^), the insulin signalling pathway (106 genes, *p* = 3.95 × 10^−5^), glucose metabolism (50 genes, *p* = 1.23 × 10^−2^), and gluconeogenesis (33 genes, *p* = 4.44 × 10^−2^); in the spleen, the target genes were significantly enriched in the T-cell receptor signalling pathway (84 genes, *p* = 1.37 × 10^−8^), the B-cell receptor signalling pathway (54 genes, *p* = 3.4 × 10^−4^), and the immune response regulated by immunoglobulin (11 genes, *p* = 1.58 × 10^−2^); in the lung, the target genes were significantly enriched in angiogenesis (163 genes, *p* = 9.76 × 10^−7^), lung development (60 genes, *p* = 1.45 × 10^−4^), and alveolar development (26 genes, *p* = 3.66 × 10^−2^); in the duodenum, the target genes were significantly enriched in the colonization of epithelial cells by bacteria (65 genes, *p* = 9.67 × 10^−5^) and positive regulation of epithelial cell proliferation (50 genes, *p* = 6.07 × 10^−4^); in the longest back muscle, the target genes were significantly enriched in skeletal muscle cell differentiation (39 genes, *p* = 2.24 × 10^−3^) and myofiber development (12 genes, *p* = 3.73 × 10^−2^).

**Figure 5:**
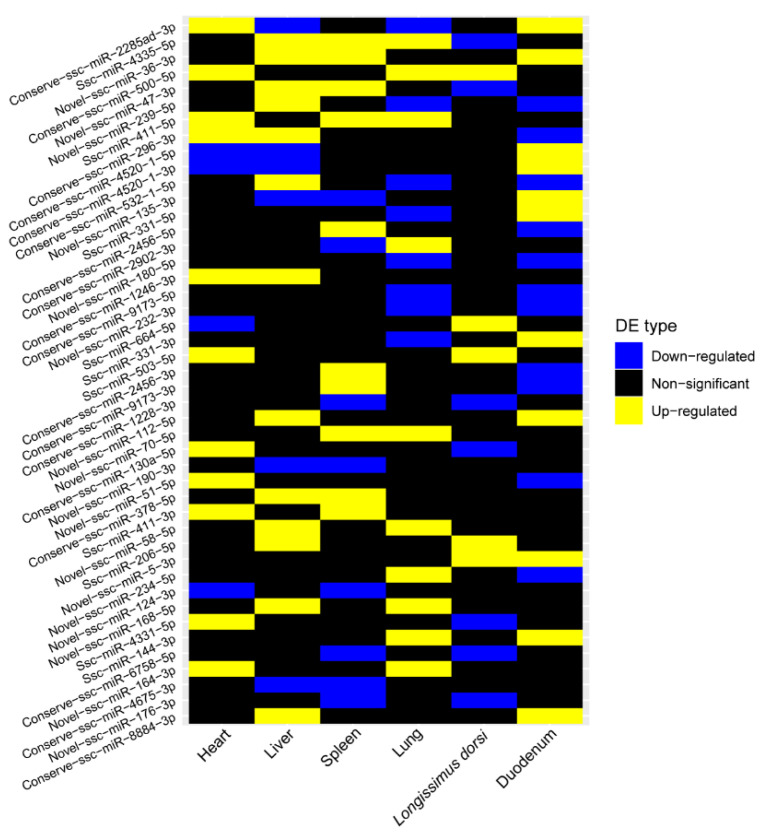
Heatmap of DE miRNAs in six tissues between prenatal and postnatal periods. Method of differential comparison: prenatal vs. postnatal.

Furthermore, we analyzed DE miRNAs that presented conserved differential patterns in at least two tissues and constructed a heatmap of their expression pattern conservation across the six tissues (Figure 5). Notably, certain DE miRNAs, such as ssc-miR-500-5p, ssc-miR-411-5p, ssc-miR-503-5p, and ssc-miR-206-5p, not only exhibited conserved differential expression patterns across tissues but also maintained consistency in their upregulation or downregulation, playing key roles in cross-tissue biological processes and postnatal adaptation. For example, miR-411 regulates myogenesis by repressing the YAF2 gene, thereby controlling the expression of myogenic factors [23]. miR-500 can suppress the expression of multiple genes and activate the NFκB signalling pathway, which promotes the proliferation and survival of gastric cancer cells. Moreover, the NFκB signalling pathway is essential for normal inflammatory and immune responses in cells [24]. When miR-503 is overexpressed in THP-1 cells, it can directly target and affect cell cycle regulatory factors, inducing G1 phase arrest [25]. miR-1228 delays stress-induced apoptosis by inhibiting the expression of MOAP1 [26]. The miR-379/miR-410 cluster plays an important role in the metabolic regulation of newborns; its knockout may lead to liver dysfunction, affecting glucose and lipid metabolism [27]. miR-206 and miR-1 regulate the fusion and differentiation of myoblasts in skeletal muscle by targeting the connexin43 (Cx43) protein [28]. miR-144 plays a role in maintaining the balance of lung fluid by targeting CFTR, which, as a chloride ion channel in lung cells, is essential for lung function [29]. miR-1246, as a target of p53, promotes apoptosis by reducing the expression of DYRK1A in the DNA damage response [30,31]. Overall, these DE miRNAs with conserved expression patterns across tissues play indispensable roles in maintaining the basic functions of prenatal tissues and adapting to the extrauterine environment after birth.

### 2.5. Regulatory Network of Differentially Expressed miRNAs in Six Tissues

**Figure 6:**
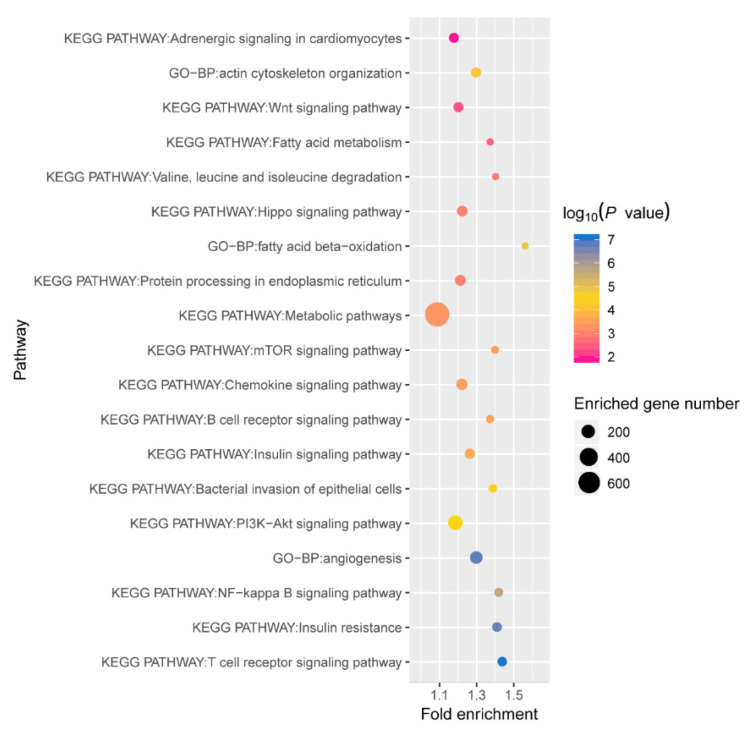
Functional enrichment of differentially expressed miRNA target genes in 6 tissues between prenatal and postnatal periods.

To elucidate the potential functions of differentially expressed miRNAs in neonatal adaptation to the extrauterine environment, we first predicted the target genes of the differentially expressed miRNAs in each tissue. We then took the intersection of target genes from the six tissues and performed functional enrichment analysis (Figure 6). The analysis results indicated that these intersecting target genes are significantly enriched in multiple biological pathways closely related to postpartum adaptability. For example, they were significantly enriched in pathways related to newborns’ response to oxygen overload and mechanical ventilation, such as angiogenesis (168 genes, *p* = 1.64 × 10^−7^), the chemokine signalling pathway (134 genes, *p* = 3.26 × 10^−6^), and the NF-kappa B signalling pathway (73 genes, *p* = 2.18 × 10^−6^). These processes affect the distal growth of the lungs and may induce the expression of proinflammatory factors in the lungs. The target genes were also significantly enriched in pathways related to neonatal metabolic adaptation, including insulin resistance (90 genes, *p* = 2.0 × 10^−7^), the insulin signalling pathway (103 genes, *p* = 2.2 × 10^−4^), metabolic pathways (776 genes, *p* = 5.0 × 10^−4^), fatty acid beta-oxidation (40 genes, *p* = 1.23 × 10^−5^), and fatty acid metabolism (39 genes, *p* = 2.97 × 10^−3^). Newborns need to adapt from the intrauterine environment, which relies on placental nutrient infusion, to the postnatal alternating phases of enteral feeding and fasting [27,32]. Furthermore, the common target genes across the six tissues were also significantly enriched in other key pathways, such as adrenergic signalling in cardiomyocytes, protein processing in the endoplasmic reticulum, degradation of branched-chain amino acids, actin cytoskeleton organization, epithelial cell bacterial colonization, the T-cell receptor signalling pathway, and the B-cell receptor signalling pathway, as well as the PI3K–Akt signalling pathway, the Hippo signalling pathway, the Wnt signalling pathway, and the mTOR signalling pathway. These pathways are closely connected to the basic functions of tissues, indicating that the shared miRNAs participate in the postnatal adaptability process of neonates through their regulatory effects.

**Figure 7:**
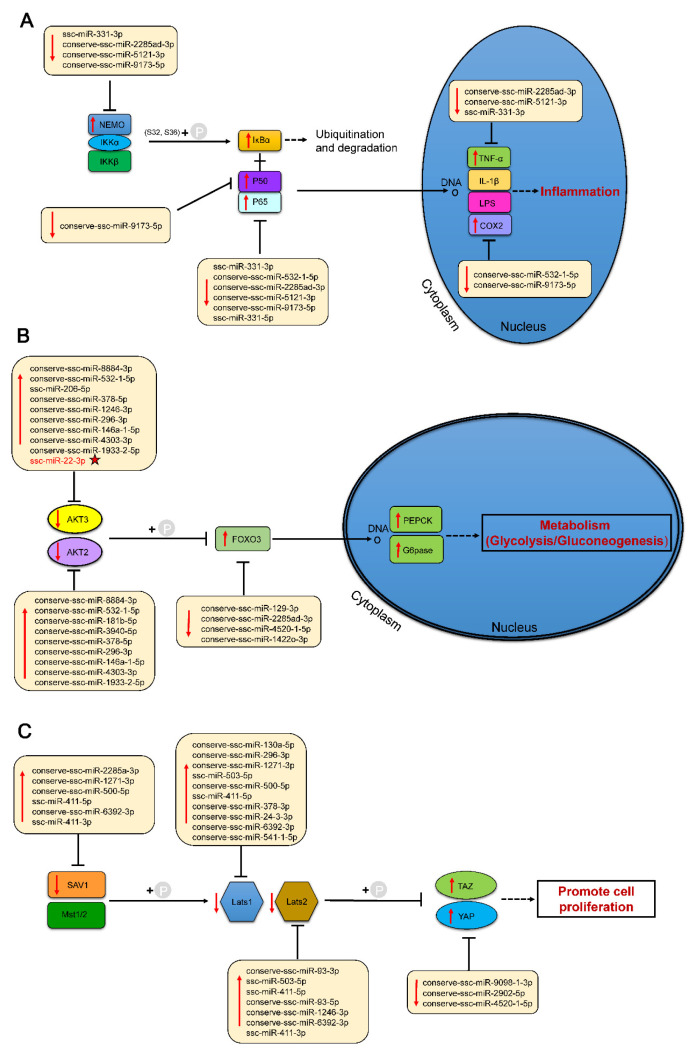
Functional enrichment pathway of target genes of differentially expressed mRNAs. (**A**) The NF-kappa B signalling pathway in the lungs. (**B**) The PI3K/AKT signalling pathway in the liver. (**C**) The Hippo signalling pathway in the heart. The up and down arrows in red indicate up- and downregulation, respectively, of DE miRNAs or target genes.

Based on the transcriptional level changes in differentially expressed miRNAs (DE miRNAs) before and after birth, their interactions with target genes (by inhibiting the expression levels of target genes), and the results of the functional enrichment analysis of target genes, we hypothesized the potential mechanisms by which these miRNAs are involved in the adaptation of piglets to the early extrauterine environment and constructed a regulatory network of miRNA–mRNA interactions. As shown in Figure 7, we highlighted three key signalling pathways that may be involved in extrauterine adaptation and their miRNA–mRNA interaction patterns.

Oxygen overload and mechanical ventilation at birth may damage the distal growth of the lungs (i.e., alveolarization) and induce the expression of pulmonary angiogenic factors (such as members of the vascular endothelial growth factor (VEGF) family) and proinflammatory factors [33,34]. The NFκB family, as a key regulator of cell proliferation, differentiation, inflammation, and angiogenesis, plays an essential role in a variety of biological processes and is particularly indispensable during the process of alveolarization [35]. We identified 73 target genes enriched in the NFκB signalling pathway across six tissues. In the lungs of newborn piglets, due to oxygen overload and mechanical ventilation at birth, the expression levels of proinflammatory factors are increased, activating the NFκB signalling pathway, among which tumour necrosis factor-α (TNF-α) derived from alveolar macrophages is essential for the activation of NFκB [36,37]. Therefore, miRNAs that target regulatory factors such as TNF-α and COX2 (PTGS2), including conserve-ssc-miR-2285ad-3p, conserve-ssc-miR-5121-3p, ssc-miR-331-3p, conserve-ssc-miR-532-1-5p, and conserve-ssc-miR-9173-5p, are significantly downregulated after birth. Moreover, the transcriptional activity of the p50 (NFKB1)/p65 (RELA) dimer is increased [37,38], and the expression levels of miRNAs targeting these transcription factors are also significantly decreased. Furthermore, the expression of the upstream NEMO gene is upregulated, whereas the expression of miRNAs targeting this gene, such as ssc-miR-331-3p, conserve-ssc-miR-2285ad-3p, conserve-ssc-miR-5121-3p, and conserve-ssc-miR-9173-5p, is significantly downregulated after birth (Figure 7A).

Delivery leads to a sudden interruption of nutrients (mainly glucose) provided by the placenta, which poses a significant metabolic challenge for newborns. To cope with this naturally occurring state of starvation, newborns first consume the glycogen stored in the liver during the late stages of embryonic development (glycogenolysis) and then activate the gluconeogenesis pathway [6]. In the functional enrichment of the intersection of target genes across six tissues, the PI3K/AKT signalling pathway was significantly enriched, with a total of 241 target genes involved in this pathway, which is involved in the activation of gluconeogenesis. In the liver, we identified 14 pairs of differentially expressed miRNAs-mRNAs that are involved in the activation of gluconeogenesis through the PI3K/AKT signalling pathway. The two main rate-limiting enzymes of gluconeogenesis in the liver, phosphoenolpyruvate carboxykinase (PEPCK) and glucose-6-phosphatase (G6Pase), regulate gluconeogenesis downstream of the PI3K/AKT signalling pathway [17]. After birth, activation of the gluconeogenesis pathway leads to significant upregulation of the expression of PCK1 (PEPCK) and G6PC (G6Pase). Accordingly, the upstream key regulatory factor, the forkhead box O transcription factor (*FoxO*), is also significantly upregulated [39,40,41]. The *FoxO* family in mammals includes *FoxO1*, *FoxO3*, and *FoxO4* [42]. In the liver, we detected the target gene *FoxO3* and its targeting miRNAs, including conserve-ssc-miR-129-3p, conserve-ssc-miR-2285ad-3p, conserve-ssc-miR-4520-1-5p, and conserve-ssc-miR-1422o-3p, which were significantly downregulated after birth. Moreover, the expression of the upstream protein *AKT* was significantly downregulated. *AKT*, as a major kinase, inhibits the activity of *FoxOs* by phosphorylating conserved sites on *FoxOs*, with specific sites including FoxO1-T24/S256/S319, FoxO3-T32/S253/S315, FoxO4-T32/S197/S262, and FoxO6-T26/S184 [43]. We found that the target genes *AKT2* and *AKT3* were both enriched in the PI3K/AKT pathway and that their targeting miRNAs, such as conserve-ssc-miR-8884-3p, conserve-ssc-miR-4335-5p, conserve-ssc-miR-532-1-5p, and conserve-ssc-miR-181b-5p, are significantly upregulated after birth (Figure 7B).

The Hippo signalling pathway is an ancient and evolutionarily conserved kinase cascade signalling pathway that regulates cell proliferation and organ size and has important biological functions [44,45]. In our study, functional enrichment analysis of target genes from six tissues revealed that a total of 109 target genes were involved in the Hippo signalling pathway. Especially in cardiac tissue, which faces potential myocardial injury that may occur at birth, the Hippo signalling pathway contributes to the regenerative capacity of the heart by promoting the proliferation of cardiomyocytes [46,47,48]. As a transcriptional cofactor of the Hippo signalling pathway, Yes-associated protein (*YAP*) plays an important role in neonatal heart regeneration and was significantly upregulated after birth. Moreover, we found that miRNAs that target the regulation of *YAP*, including conserve-ssc-miR-9098-1-3p, conserve-ssc-miR-2902-5p, and conserve-ssc-miR-4520-1-5p, were significantly downregulated after birth. The upstream negative regulatory factors of *YAP*, such as *Lats1/2* and *Mst1/2* [49], as well as *SAV1*, an auxiliary protein for *Mst1/2* [50,51], were also significantly downregulated. The target genes *SAV1*, *Lats1*, and *Lats2* that we detected are enriched in the Hippo signalling pathway, and the miRNAs that target these genes, such as conserve-ssc-miR-2285ad-3p, conserve-ssc-miR-1271-3p, conserve-ssc-miR-500-5p, and ssc-miR-411-5p, were significantly upregulated after birth (Figure 7C).

### 2.6. Re-Establishment of Glucose Homeostasis in Neonatal Piglets

**Figure 8:**
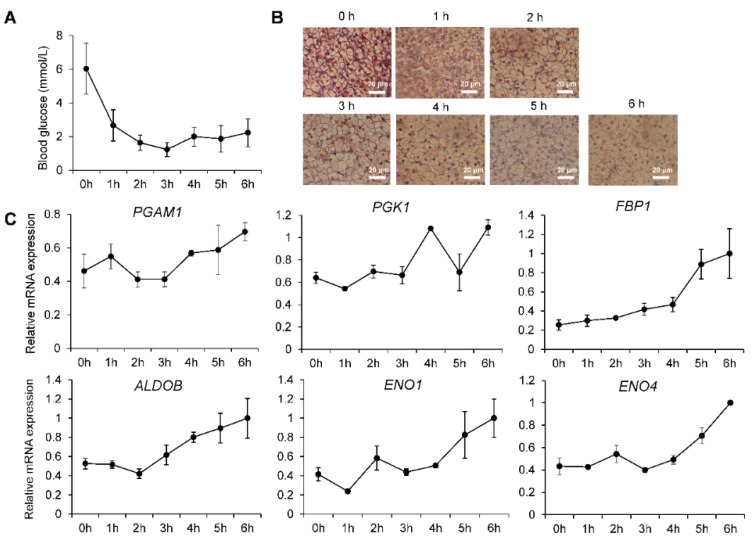
Glycemic homeostasis of newborn piglets. (**A**) Blood glucose measurement of newborn piglets. (**B**) PAS staining of newborn pig liver slices. (**C**) qRT-PCR of gluconeogenesis genes in newborn piglets.

To explore the role of miRNAs in glucose metabolism in the livers of neonatal piglets, we continuously monitored blood glucose levels in neonatal piglets (from 0 to 6 h) (Figure 8A). The initial blood glucose concentration in neonatal piglets was 6.03 mmol/L. Due to the disconnection from the placental energy supply after birth, the blood glucose concentration rapidly declined, reaching its lowest point three hours after birth (1.23 mmol/L), then gradually rebounded, stabilizing at 2.23 mmol/L six hours after birth. This change indicates that piglets establish a new glucose homeostatic mechanism within approximately 6 h after birth.

To understand the molecular mechanism of this regulatory process, we assessed the glycogen content in the livers of piglets using periodic acid–Schiff (PAS) staining of liver sections (Figure 8B). The assessment revealed that the glycogen content in the livers of piglets continued to decline after birth, reaching its lowest level six hours after birth. This finding indicates that in order to maintain glucose levels and energy supply in the body, the stored glycogen in the liver is rapidly mobilized to replenish blood glucose in neonatal piglets.

**Figure 9:**
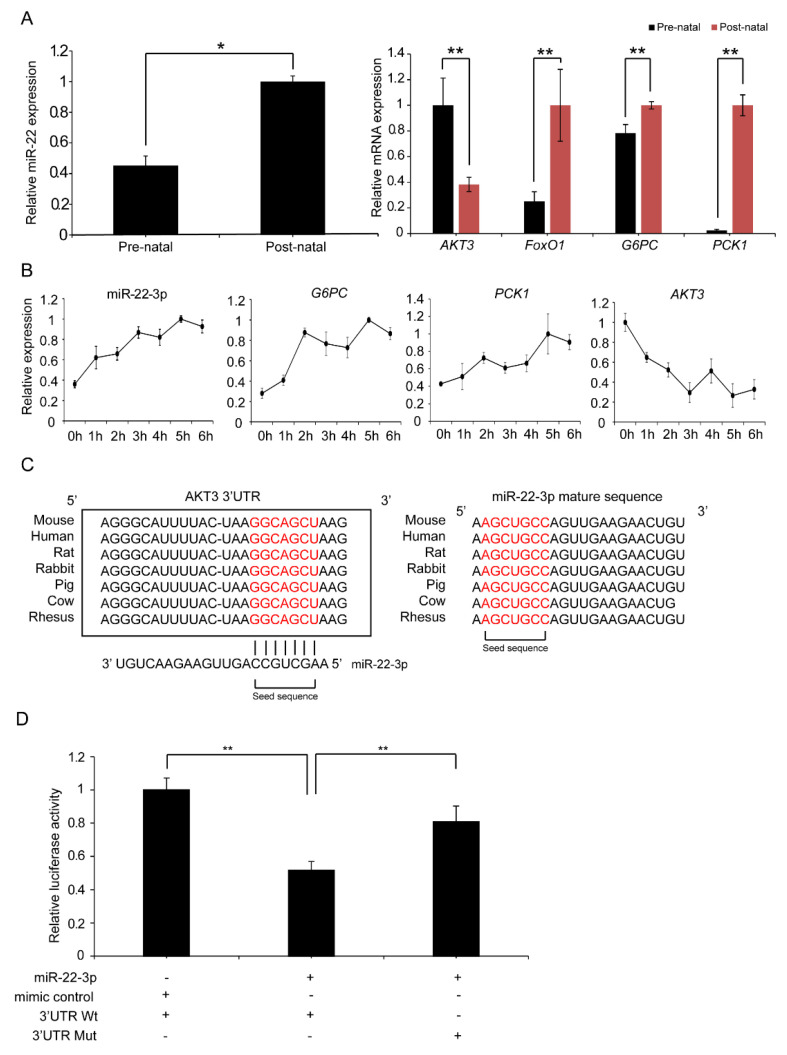
qRT-PCR of miR-22-3p and genes related PI3K/AKT signalling pathway in piglet liver; miR-22-3p targets AKT3 by binding to its 3′UTR. (**A**) qRT-PCR of miR-22-3p and AKT3/FoxO1/PCK1/G6PC levels in prenatal and postnatal piglet liver. (**B**) qRT-PCR of liver mir-22-3p and AKT3/PCK1/G6PC in newborn piglets. (**C**) miR-22-3p binding site on AKT3 3′UTR and its conservation across species. (**D**) Luciferase activity of miR-22-3p and AKT3 3′UTR (Wt/Mut). “Pre” and “Post” represent “prenatal” and “postnatal”, respectively. Three independent experiments performed in triplicate, and all data are expressed as mean ± SD. * *p* < 0.05; ** *p* < 0.01.

Furthermore, we determined the expression levels of key gluconeogenic genes, including *G6PC*, *PCK1*, *PGAM1*, *PGK1*, *FBP1*, *ALDOB*, *ENO1*, and *ENO4*, in the livers of piglets via real-time quantitative PCR technology (Figure 8C and Figure 9B). The expression levels of these gluconeogenesis-related genes continued to rise after birth in piglets. This finding indicates that neonatal piglets not only mobilized the stored glycogen in the liver but also increased the gluconeogenic capacity of the liver to convert noncarbohydrate substances into glucose, thereby maintaining glucose homeostasis.

### 2.7. Validation of the Targeting Relationship Between AKT3 and miR-22-3p in the Liver

In the functional enrichment analysis of differentially expressed miRNA (DE miRNA) target genes described above, we found that miR-22-3p is involved in the PI3K/AKT signalling pathway in the liver, which activates gluconeogenesis. The sequencing data indicated that the expression of miR-22-3p is significantly upregulated after birth in neonatal piglets. We hypothesize that miR-22-3p may suppress the expression of its target gene AKT3, thereby reducing the phosphorylation level of FoxO1, thus increasing the expression levels of the key gluconeogenic enzymes PCK1 and G6PC, promoting gluconeogenesis, and helping to maintain glucose homeostasis in neonatal piglets. To investigate this hypothesis, we measured the expression levels of miR-22-3p, AKT3, FoxO1, PCK1, and G6PC in liver samples from piglets before and after birth via qRT–PCR. miR-22-3p was significantly upregulated in liver samples after birth, whereas AKT3 was significantly downregulated, and the expression of FoxO1, PCK1, and G6PC was significantly upregulated (Figure 9A). In addition, we monitored the expression levels of miR-22-3p, AKT3, G6PC, and PCK1 in neonatal piglets from 0 to 6 h after delivery. The expression of miR-22-3p continued to increase, the expression level of AKT3 continued to decrease, and the expression of the gluconeogenic genes G6PC and PCK1 also continued to increase (Figure 9B), further supporting the hypothesis that miR-22-3p inhibits the expression of AKT3 and promotes gluconeogenesis.

To validate the relationship between miR-22-3p and AKT3, we used the online software TargetScan 7.1 to predict the target genes of miR-22-3p and identified AKT3 as a target gene. We found that nucleotides 2004–2011 in the 3′UTR of AKT3 are the binding sites for the seed sequence of miR-22-3p, and this binding site is conserved across multiple species (Figure 9C). Furthermore, we cloned the wild-type (Wt) and mutant (Mut) 3′UTR sequences of AKT3 downstream of Luc2 in the pmirGLO vector to construct a dual-luciferase reporter vector. These recombinant vectors were co-transfected into HeLa cells with the miR-22-3p mimic or mimic control, and the luciferase activity was detected after 48 h. The miR-22-3p mimic significantly reduced the luciferase activity of pmirGLO-AKT3 with the wild-type binding site (*p* < 0.05), whereas the luciferase activity with the mutant binding site was unaffected (Figure 9D). These results support that miR-22-3p interacts with the binding site in the 3′UTR of AKT3 through its seed sequence and inhibits the expression level of AKT3 within cells.

### 2.8. miR-22-3p Promotes Gluconeogenesis by Repressing AKT3 to Regulate the PI3K/AKT Signalling Pathway

**Figure 10:**
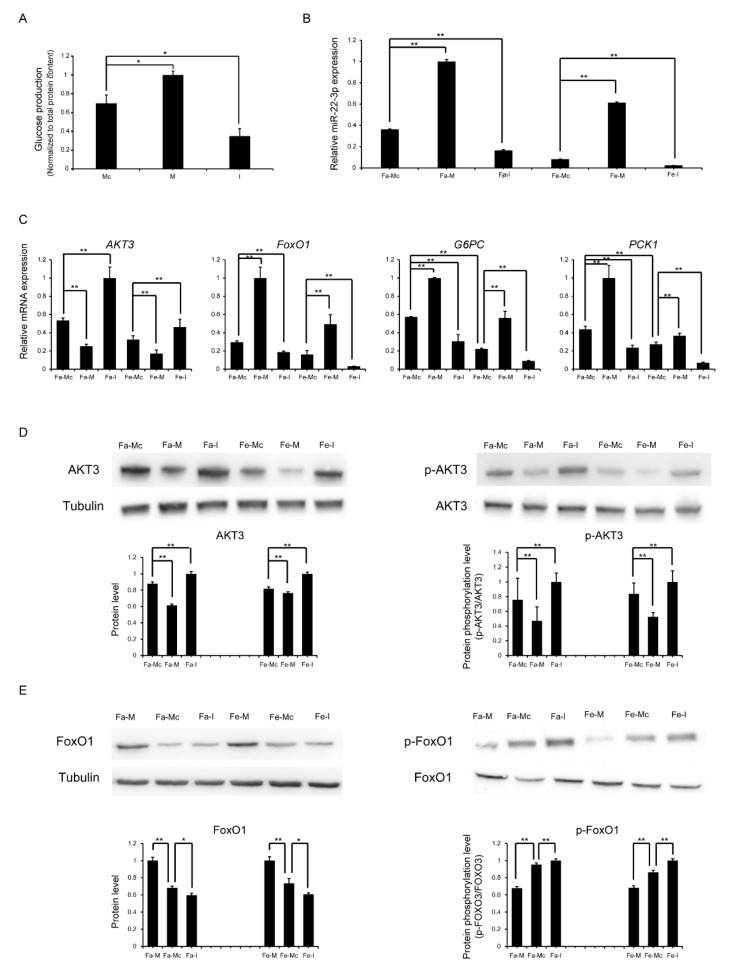
The impact of miR-22-3p on PI3K/AKT signalling and gluconeogenesis. (**A**) HepG2 cells were transfected with mimic control, miR-22-3p mimic, and inhibitor; the amount of glucose produced in the culture medium after incubation in glucose-free and phenol red-free medium; expression levels of miR-22-3p after HepG2 cells were transfected with mimic control, miR-22-3p mimic, and inhibitor, and incubated in complete and serum-free media. (**B**) Expression levels of miR-22-3p. (**C**) Protein levels of AKT3 and phospho-AKT3, and (**D**) FoxO1 and (**E**) phospho-FoxO1. “Fa”, “Fe”, “Mc”, “M”, and “I” represent “fasting”, “non-fasting”, “mimic control”, “mimic”, and “inhibitor”, respectively. Three independent experiments were performed with three replicates each; data are presented as mean ± SD; * *p* < 0.05; ** *p* < 0.01.

The liver has a unique ability to both produce and consume glucose. Newborns suddenly lose the glucose supplied by the placenta at birth. To cope with starvation and hypoglycaemia, they initially break down stored liver glycogen while simultaneously activating the gluconeogenesis pathway [6] in the liver. The seed sequence of miR-22-3p and its binding site with the target gene AKT3 are conserved across species (Figure 9C). We selected HepG2 cells to verify the role of miR-22-3p in promoting the hepatic gluconeogenesis mechanism. The overexpression or inhibition of miR-22-3p in HepG2 cells was achieved by transfecting the cells with the miR-22-3p mimic or inhibitor. After 24 h, we replaced the culture medium with a medium that did not contain glucose or phenol red and contained 20 mM sodium lactate and 2 mM sodium pyruvate to simulate gluconeogenic conditions. After 3 h, we measured the glucose content in the culture medium. When miR-22-3p was overexpressed, the glucose content significantly increased, indicating enhanced gluconeogenesis, whereas the inhibition of miR-22-3p resulted in the opposite effect, confirming that miR-22-3p can promote gluconeogenesis (Figure 10A).

Furthermore, to explore whether miR-22-3p activates gluconeogenesis through the PI3K/AKT signalling pathway, we overexpressed or inhibited miR-22-3p in HepG2 cells. After 24 h, the culture medium was replaced with serum-free medium to simulate the postnatal fasting state (complete medium containing 10% fetal bovine serum was used for the control group), and after an additional 12 h of culture, total RNA was extracted, and the expression levels of AKT3, FoxO1, and the gluconeogenic genes PCK1 and G6PC were detected via qRT–PCR. The expression level of miR-22-3p was significantly upregulated or downregulated after transfection with the miR-22-3p mimic and inhibitor, respectively (Figure 10B). In the experimental group without miR-22-3p transfection, that is, with mimic control transfection, the expression of PCK1 and G6PC in the fasting group was significantly greater than that in the non-fasting control group (Figure 10C), further confirming the activation of gluconeogenesis under fasting conditions. Additionally, in both the fasting and control groups, the expression of the AKT3 gene was significantly downregulated when miR-22-3p was overexpressed, while the expression of the FoxO1 gene was significantly upregulated, along with that of PCK1 and G6PC; the opposite results were observed with the use of the inhibitor (Figure 10C). AKT3, a key kinase in the PI3K/AKT signalling pathway, inhibits the activity of FoxO1 through its phosphorylation. Overexpression of miR-22-3p led to a significant downregulation of AKT3 protein levels, along with a significant decrease in pAKT3 protein levels; this resulted in a significant downregulation of pFoxO1 protein levels, while the FoxO1 protein levels were significantly upregulated (Figure 10D,E). The opposite results were observed with the use of the inhibitor. These results suggest that miR-22-3p can upregulate the expression of FoxO1 by targeting AKT3, thereby increasing the expression of the key rate-limiting enzymes PCK1 and G6PC in gluconeogenesis and promoting gluconeogenesis. Together, our experimental results show that the expression of miR-22-3p in the liver of piglets after birth was significantly upregulated, which promoted gluconeogenesis by inhibiting the expression of the target gene AKT3 through the PI3K/AKT signalling pathway, increasing glucose production, and maintaining glucose homeostasis in the body. However, these speculations still require further experimental validation to determine their specific role in gluconeogenesis.

## 3. Discussion

miRNAs, as essential regulators of gene expression, play key roles in tissue development and homeostasis. Given the physiological and pathological similarities between pigs and humans and the convenience of experimental manipulation, pigs are widely used as mammalian models for studying human diseases. This study analyzed the miRNA expression patterns in six tissues of piglets 24 h before birth and 6 h after birth (heart, liver, spleen, lung, multifidus muscle, and duodenum) through small RNA sequencing and identified 223 differentially expressed miRNAs, with the most significant differences observed in the duodenum.

Through tissue-specific analysis, we identified miRNAs that are differentially expressed specifically in each tissue, and comparisons with miRNA expression data from humans and rats revealed that the miRNA expression patterns in pigs are highly consistent with those in humans and rodents [52], confirming the reliability of pigs as research models. Specifically, the muscle-specific miRNAs miR-1-3p and miR-206 were differentially expressed in the multifidus muscle [53,54,55], further validating the accuracy of these findings.

This study thoroughly explored the role of miRNAs in the early extrauterine adaptation and late development of newborns. In the lungs, miRNAs are involved in the regulation of inflammatory responses through the NFκB signalling pathway, which helps newborns adapt to a hyperoxic environment. In the liver, miR-22-3p promotes gluconeogenesis through the PI3K/AKT signalling pathway, increasing glucose production and maintaining blood glucose homeostasis. In the heart, miRNAs may affect the proliferation of cardiomyocytes and organ size through the Hippo signalling pathway. However, these hypotheses still need to be validated through more experiments, particularly in vivo studies, to confirm their actual functions in the organs of neonatal piglets.

The liver plays an essential role in the neonatal adaptation to the extrauterine environment, particularly in maintaining glucose homeostasis. Studies have shown that miRNAs regulate key genes such as AKT3 and FoxO1 to promote gluconeogenesis, ensuring that neonates maintain energy balance in the short term after weaning. This process is critical for the adaptation of neonatal energy metabolism.

miRNAs play a role in multiple aspects of neonatal adaptation to the extrauterine environment. Specifically, miR-22-3p regulates the gluconeogenesis process in the liver, ensuring that neonates transition from maternal nourishment to self-sustaining energy balance. Additionally, miRNAs may play a key role in the adaptive development of the heart by regulating the Hippo signalling pathway, helping the heart complete regeneration and development during the early postnatal stage. These findings provide new insights into the role of miRNAs in the health and disease of newborns and offer valuable information for future research and therapy.

This study, through a piglet model, reveals the important role of miRNAs in the adaptation of newborns to the extrauterine environment and the developmental process. miRNAs affect the function of organs such as the lungs, liver, and heart of newborns through different signalling pathways, such as the NFκB, PI3K/AKT, and Hippo pathways. These findings not only enrich our understanding of the adaptation mechanisms of newborns to the extrauterine environment but also provide new ideas for the development of potential therapeutic tools for the treatment of related diseases.

The study also revealed that the miRNA–mRNA interaction network promotes NFκB signal activation in the lungs of newborns, enhances inflammatory responses, and is beneficial for the adaptation of newborn piglets to the extrauterine environment. In the liver, miRNAs regulate hepatic gluconeogenesis through the PI3K/AKT signalling pathway, increasing blood glucose levels, which is essential for maintaining the homeostasis of energy levels in newborns in the short period after birth. The risk of neonatal mortality is very high from the time of delivery until neonates can feed. Therefore, sugar homeostasis regulation mediated by the miR-22-3p-AKT3-PI3K/AKT regulatory axis may be a key link in the establishment of extrauterine environmental adaptability in newborns.

The Hippo signalling pathway may play an important role in the neonatal heart’s adaptation to the extrauterine environment by regulating cell proliferation and organ size. Its potential function in cardiac regeneration offers a new perspective and helps us better understand the adaptive changes in the neonatal heart after birth.

Glucose is the primary energy source for mammals, and maintaining glucose homeostasis is one of the key mechanisms for the survival of organisms. In mammals, the liver is a key organ for maintaining glucose homeostasis; it regulates the processes of glucose uptake and storage and controls the balance of plasma and hepatic glucose levels through glucose output [56,57]. Under fasting conditions, the production of sufficient glucose by the liver is essential for maintaining the basic functions of other tissues [58]. In the early stages of fasting, the liver releases glucose by enhancing glycogenolysis, whereas in the later stages of fasting, a prolonged state of hypoglycaemia induces the liver to synthesize glucose de novo, a process known as gluconeogenesis.

Transcriptional regulation of gluconeogenesis has been shown to be a potential target for blood glucose control via type 2 diabetes medications. Therefore, understanding the complex regulatory mechanisms of the liver’s gluconeogenic transcriptional circuitry is essential for developing potential therapeutic tools for the treatment of this disease [58]. In recent years, many miRNAs, such as miR-33b [59], miR-696 [60], miR-122 [61], miR-451 [62], miR-137 [63], and miR-423-5p [64], among others, have been shown to be involved in the regulation of hepatic gluconeogenesis and affect hepatic gluconeogenesis and lipid metabolism through various mechanisms.

In summary, miRNAs play important roles in the adaptation of newborns to the extrauterine environment and the developmental process. By regulating different signalling pathways, miRNAs affect the function of organs such as the lungs, liver, and heart of newborns, promoting their adaptation to the extrauterine environment. Additionally, miRNAs may have significant clinical application value in the postnatal adaptation process. As research advances, we are gradually recognizing the potential role of miRNAs in early feeding management. By regulating the expression of relevant genes, miRNAs may help improve gut development, promote immune system maturation, and optimize feeding strategies to enhance the health of neonatal animals. Specifically, miRNAs could serve as biomarkers to help predict and assess potential health risks during the postnatal adaptation process in neonate pigs, providing opportunities for early clinical intervention. Furthermore, miRNAs may play an important role in health protection strategies, especially in regulating the gut microbiota and enhancing immune responses through nutritional interventions. These findings not only provide new insights into the role of miRNAs in neonatal health and disease but also offer valuable information for future research and therapies.

## 4. Conclusions

The miRNA expression patterns in piglets before and after birth are not conserved across different tissues. Most differentially expressed miRNAs (DE miRNAs) are differentially expressed in no more than four tissues simultaneously. These tissue-specific miRNAs are significantly enriched in pathways related to maintaining the functions of their respective tissues, whereas miRNAs with conserved expression patterns across multiple tissues play a key role in tissue development and postnatal adaptation. Particularly in the liver, miR-22-3p is significantly upregulated after birth and may be involved in the PI3K/AKT signalling pathway through its target gene AKT3, promoting gluconeogenesis and helping newborns regulate blood sugar levels. In the first few hours after birth, newborn piglets rely on liver-stored glycogen and the conversion of noncarbohydrate substances to glucose through gluconeogenesis to maintain glucose homeostasis. In HepG2 cells, the overexpression of miR-22-3p inhibits the AKT3 gene and activates the PI3K/AKT pathway, thereby increasing glucose production. These findings indicate that miRNAs play important roles in the adaptation of newborns to the extrauterine environment, especially in the adaptation of key organs such as the lungs, liver, and heart under conditions such as early hyperoxia, hunger, hypoglycaemia, and changes in oxygen and stress.

## 5. Materials and Methods

### 5.1. Experimental Animal Samples

The experimental animals were a cross between Tibetan pigs and Meishan pigs sourced from the Poly Pig Farm in Chengdu, Sichuan Province. We selected three female piglets 24 h before birth (at 113 days of gestation, 24 h before the expected delivery date) and three other female piglets within 6 h after birth (without having ingested colostrum). The pre-birth piglet samples were rapidly obtained from the sows via caesarean section. To strengthen the accuracy of the experiment, we selected three female piglets with similar body weights. Samples were taken from the piglets’ hearts, livers, spleens, lungs, longest back muscles, and duodenum. After the samples were obtained, they were rapidly frozen in liquid nitrogen and then stored at −80 °C for subsequent experimental use. The research procedures involving animals were conducted in accordance with the “Regulations on the Administration of Experimental Animals” (revised by the Ministry of Science and Technology of China in June 2004) and were approved by the Institutional Animal Care and Use Committee of the College of Animal Science and Technology at Sichuan Agricultural University in Sichuan Province, China, with licence number DKY-S20163658.

### 5.2. Total RNA Extraction from Tissues and Cells

Samples from different tissues (25 mg each for heart and liver, 10 mg for spleen, and 30 mg for muscle) and cultured cells were lysed with 1 mL of TRIzol each. After lysis, the mixture was centrifuged at 4 °C and 12,000× *g* for 5 min to separate the supernatant. Chloroform and isopropanol were added to the supernatant, and the mixture was centrifuged separately to precipitate the RNA. The RNA pellet was washed with 75% ethanol, dried, and then dissolved in DEPC water. The dissolved RNA was immediately checked for quality and used for experiments or stored at −80 °C to avoid repeated freezing and thawing.

### 5.3. Total RNA Quality Control

To ensure the quality of the small RNA library, a rigorous quality control of the extracted total RNA was performed as follows:

(1) Purity assessment of total RNA: A NanoDrop ND-2000 spectrophotometer was used to measure the absorbance of the total RNA sample at 260 nm and 280 nm and calculate the A260/A280 ratio. A ratio close to 2.0 (with a generally acceptable range between 1.9 and 2.1) indicated a qualified sample.
(2) Integrity assessment of total RNA: The total RNA sample was assessed by 1.2% denaturing agarose gel electrophoresis, with an intensity ratio of the 28S band to the 18S band of approximately 2 and the absence of smears and trailing indicating a qualified sample.

### 5.4. Small RNA Library Construction and Sequencing

The small RNA library was constructed via the Illumina TruSeq Small RNA Prep Kit (San Diego, CA, USA), and quality control was performed via the Agilent 2200 TapeStation. The RNA fragments were separated via gel electrophoresis; fragments 50 bp in length were collected, and the fragments were repurified after Illumina sequencing adapters were added to both ends. Single-stranded cDNA was generated through reverse transcription, and the library was established after two rounds of PCR amplification. Sequencing was performed using the Illumina HiSeq 2500 platform and processed with CASAVA 1.8 software to generate raw reads in FASTQ format.

### 5.5. miRNA Data Analysis

After quality filtering to remove low-quality sequences, duplicated sequences, and adapters, clean reads were obtained. These clean reads were aligned and analyzed using the mRNA, RFam, and Repbase databases and precisely matched with the pig ARS1 genomic sequence. Mappable reads were selected using Bowtie [65], and miRNAs were identified via miRDeep (version 2.0.0.7) [66] and the miRbase 21 database [67]. The selection criterion was miRNAs with a read count of at least 3 in at least one sample. The read counts of these miRNAs were normalized to the RPM for comparison. Finally, clustering and PCA were performed by calculating z-scores to standardize the data.

### 5.6. Tissue-Specific Analysis

To assess the tissue-specific expression patterns of miRNAs, we used TSI, a quantitative measure ranging from 0 to 1, with higher values indicating stronger tissue specificity of the miRNA. miRNAs with a TSI below 0.15 are considered “housekeeping genes”, whereas those with a TSI above 0.85 are defined as “tissue-specific”. The TSS, which is based on Shannon entropy, quantifies the similarity between the miRNA expression pattern and the extreme pattern of being expressed in only one tissue. The TSS values for each miRNA across different tissues were calculated, and for miRNAs with a TSI above 0.85, the tissue with the highest TSS value was selected as the tissue where it was specifically expressed.

### 5.7. Differential miRNA Identification

To reveal the differences in the miRNA transcriptome before and after birth, differentially expressed (DE) miRNAs were identified using edgeR software (V.4.4.1) [68]. To obtain the normalized expression levels, read counts were imported into edgeR and normalized using the TMM (trimmed-mean-of-M-values) algorithm. Differential miRNAs were defined as those with a fold change > 1.5 in expression levels before and after birth and a Benjamini–Hochberg false discovery rate (FDR) < 0.05.

### 5.8. miRNA Target Gene Prediction and Functional Enrichment

Differential miRNAs before and after birth were extracted, and their target genes were predicted via TargetScan 7.0. By leveraging the homologous genes available in the ENSEMBL database, pig target genes were converted into their human homologues. Gene Ontology (GO) and pathway analyses were subsequently conducted using the online software DAVID (V.2023q3) to identify the potential biological processes and signalling pathways associated with the differentially expressed miRNAs.

### 5.9. Dual-Luciferase Assay to Verify Target Relationships

The miR-22-3p sequence was obtained from miRBase (http://www.mirbase.org/ accessed on 4 December 2023), and the AKT3 gene sequence was downloaded from NCBI (https://www.ncbi.nlm.nih.gov/ accessed on 4 December 2023). RNAhybrid (https://bibiserv.cebitec.uni-bielefeld.de/rnahybrid/ accessed on 5 March 2024) was used to predict the binding site of miR-22-3p in the AKT3 3′ untranslated region (3′-UTR). Based on this, wild-type (Wt) and mutant (Mut) sequences of the AKT3 3′-UTR were designed. A mutation was introduced at the binding site of the Mut sequence using site-directed mutagenesis.

AKT3 3′-UTR Wt sequence: 5′-GAGCTCGGGTTCAAGGGCATTTTACTAAGGCAGCTAAGACATATGCAGACATAGATCTCGAG-3′.

AKT3 3′-UTR Mut sequence: 5′-GAGCTCGGGTTCAAGGGCATTTTACTAATAGCTAGAAGACATATGCAGACATAGATCTCGAG-3′.

The sequences were synthesized by Qingke Zixi Biotechnology (Chengdu, China) and cloned into pmirGLO vectors. The vector dry powder (4 μg) was resuspended in 40 μL of deionized water to obtain a concentration of 100 ng/μL.

The miR-22-3p mimic and mimic control were synthesized by RiboBio Co., Ltd. (Guangzhou, China), and reconstituted in 250 μL of sterile DEPC water to a final concentration of 20 μM.

HeLa cells were co-transfected with the pmirGLO vector (Wt/Mut) and miR-22-3p mimic or mimic control using Lipofectamine™3000 (Thermo Fisher Scientific, Waltham, MA, USA). After 48 h, luciferase activity was measured using the Dual-Glo^®^ Luciferase Assay System (Promega Corporation, Madison, WI, USA). The data were normalized by Renilla luciferase activity and expressed relative to the mimic control.

### 5.10. HepG2 Cell Culture and Transfection

HepG2 cells, obtained from the Stem Cell Bank of the Chinese Academy of Sciences, were cultured in fresh medium containing 10% fetal bovine serum once they reached 60% confluence, and the culture was continued at 37 °C in a 5% CO_2_ environment. Upon reaching 80% confluence, the cells were passaged, which included disposal of the old medium, washing with PBS, trypsin digestion, termination of digestion, centrifugation, resuspension, and replating. After being cultured in a 6-well plate to a density of 50%, cells in the logarithmic growth phase were transfected. Twenty-four hours post-transfection, the cells were divided into a starvation group, which was switched to serum-free high-glucose DMEM, and a normal group with medium containing 10% fetal bovine serum, and further cultured for 12 h. The cells were subsequently collected for the extraction of RNA and proteins.

### 5.11. Reverse Transcription and Quantitative Real-Time PCR (qRT-PCR)

Total RNA from cells and tissues was reverse-transcribed into miRNA and mRNA using the Takara PrimeScript^®^ and SYBR^®^ PrimeScript™ kits (Kusatsu, Japan), following the manufacturer’s protocols. For mRNA reverse transcription, the reaction conditions were as follows: 42 °C for 2 min, 37 °C for 15 min, and by inactivation at 85 °C for 5 s. For miRNA reverse transcription, the conditions were 37 °C for 60 min, followed by inactivation at 85 °C for 5 s. After reverse transcription, RNase-free water was added to adjust the final volume to 100 μL, and the samples were stored at −20 °C.

Primers for quantitative RT-PCR (qRT-PCR) were designed using NCBI Primer Blast. For mRNA, primers were designed based on the coding sequence (CDS) regions obtained from NCBI, while for miRNA, primers were designed based on mature miRNA sequences downloaded from miRBase. All primers were synthesized by Qingke Zixi Biotechnology Co., Ltd.

qRT-PCR was performed using Takara SYBR Premix Ex Taq II (2×) reagent according to the manufacturer’s instructions. The data were analyzed using the 2^−△△Ct^ method, and normalization was performed using GAPDH for mRNA and U6 for miRNA as internal control genes. Melting curve analysis was performed to confirm the specificity of the primers and ensure that no non-specific amplification occurred.

### 5.12. Western Blot

Total protein was extracted from samples using Biyuntian lysis buffer (without inhibitors), and the concentration was determined using a BCA protein assay kit according to the manufacturer’s instructions. After denaturation, protein samples were mixed with 4× sample buffer and 2-mercaptoethanol, boiled for 5 min, then placed on ice for later use. A precast gel was placed into the electrophoresis chamber, and buffer was added. The samples were loaded based on their concentration, and a protein marker was included to estimate the molecular weight of the proteins. Electrophoresis was performed at a constant voltage of 200 V until the protein bands were fully separated. For transfer, PVDF membranes and filter paper were prewetted in methanol and transfer buffer, respectively, and the membrane was transferred using a semidry transfer apparatus for 25 min at 15 V and 0.9 A. After blocking with 5% non-fat dry milk in TBST for 2 h at room temperature, membranes were incubated with primary antibodies diluted in blocking buffer for 2 h at room temperature or overnight at 4 °C. Following incubation, membranes were washed three times with TBST. The membranes were then incubated with secondary antibodies for 2 h at room temperature. After additional washing, proteins were detected by enhanced chemiluminescence (ECL), and images were captured using a Bio-Rad ChemiDoc™ XRS+ imaging system (Hercules, CA, USA). The specific primary and secondary antibodies used, as well as their concentrations, should be clearly stated for reproducibility. Quantification of protein expression can be performed by normalizing to an internal control protein (e.g., GAPDH or β-actin).

### 5.13. Glucose Production Assay

Fifty millilitres of glucose production medium was prepared. This contained 20 mM sodium lactate and 2 mM sodium pyruvate. HepG2 cells were cultured in a 12-well plate to 60% confluence and then transfected with the miR-22-3p mimic, inhibitor, or mimic control, with three replicates for each group. After 24 h of transfection, the medium was replaced with glucose production medium, and the cells were cultured for an additional 3 h. The culture supernatant was collected, and the glucose content was measured. The absorbance was determined via a microplate reader, and the data were normalized on the basis of protein content.

### 5.14. Statistical Analysis

In this study, all the experiments were performed in triplicate, with three independent replicates. All the data are presented as the means ± standard deviations. The statistical significance between two groups of data was calculated using a t test, whereas comparisons among more than two groups were calculated using one-way analysis of variance (ANOVA). The calculations were performed using SPSS 19.0 software. A *p* value less than 0.05 indicates a significant difference, and a *p* value less than 0.01 indicates a highly significant difference.

## Figures and Tables

**Table 1:** Mature miRNAs and miRNA precursors identified in this study.

Type	Pre-miRNAs	miRNA-5p	miRNA-3p	Both	Mature miRNAs
Known	239	21	13	205	444
Conserved	482	129	138	215	697
Novel	250	53	77	120	370
Total	971	203	228	540	1511

**Table 2:** Number of DE miRNAs between pre- and postnatal populations within each tissue.

Tissues	Heart	Lung	Spleen	Muscle	Liver	Duodenum	Total
Number of DE miRNAs	34	38	33	21	37	60	223

**Table 3:** Number of DE miRNAs between pre- and postnatal populations overlapping among tissues.

Number of Tissues	1	2	3	4	5	6
Number of overlapping DE miRNAs	119	33	10	2	0	0

## Data Availability

The data presented in this study are available upon request from the corresponding author.

## Supplementary Materials

The following supporting information can be downloaded at: https://www.mdpi.com/article/10.3390/ijms26031316/s1.
