# Supplementary material for: Identification and Functional Analysis of Key microRNAs in the Early Extrauterine Environmental Adaptation of Piglets
Source: Int J Mol Sci. 2025 Feb 4;26(3):1316. doi: 10.3390/ijms26031316 (PMC11818927; doi:10.3390/ijms26031316)
Supplement: Supplementary file 1 [file ijms-26-01316-s001.zip › ijms-3434790-supplementary.pdf]

Supplementary Table S1. DEmiRNAs in different tissues.

Genes	LogFC	LogCPM	LR	PValue	FDR	Tissue
conserve-ssc-miR-1246-3p	-8.73	2.09	13.52	2.36E-04	1.27E-02	liver
conserve-ssc-miR-129-3p	9.26	2.58	16.89	3.95E-05	2.84E-03	liver
conserve-ssc-miR-1422o-3p	8.12	1.52	12.13	4.97E-04	2.28E-02	liver
conserve-ssc-miR-146a-1-5p	-8.40	1.79	12.27	4.60E-04	2.17E-02	liver
conserve-ssc-miR-181b-5p	-8.16	1.57	11.39	7.38E-04	3.18E-02	liver
conserve-ssc-miR-18a-3p	6.12	4.24	13.42	2.49E-04	1.30E-02	liver
conserve-ssc-miR-1933-2-5p	-8.86	2.21	14.07	1.76E-04	9.88E-03	liver
conserve-ssc-miR-2285ad-3p	13.24	6.50	42.44	7.30E-11	2.20E-08	liver
conserve-ssc-miR-296-3p	-12.71	5.97	36.42	1.59E-09	2.40E-07	liver
conserve-ssc-miR-378-5p	-10.16	3.46	20.31	6.57E-06	6.62E-04	liver
conserve-ssc-miR-3940-5p	-8.16	1.57	11.39	7.38E-04	3.18E-02	liver
conserve-ssc-miR-4303-3p	-9.22	2.55	15.62	7.76E-05	5.08E-03	liver
conserve-ssc-miR-4520-1-3p	16.74	9.99	76.69	2.00E-18	3.02E-15	liver
conserve-ssc-miR-4520-1-5p	14.59	7.84	55.28	1.04E-13	7.89E-11	liver
conserve-ssc-miR-4675-3p	9.49	2.80	18.03	2.18E-05	1.64E-03	liver
conserve-ssc-miR-532-1-5p	-9.43	2.75	16.59	4.63E-05	3.18E-03	liver
conserve-ssc-miR-646-3p	-8.86	2.21	14.07	1.77E-04	9.88E-03	liver
conserve-ssc-miR-8884-3p	-13.01	6.27	38.71	4.92E-10	9.29E-08	liver
novel-ssc-miR-106-3p	-13.21	6.47	40.30	2.18E-10	5.48E-08	liver
novel-ssc-miR-112-5p	-10.25	3.54	20.75	5.23E-06	5.64E-04	liver
novel-ssc-miR-135-3p	12.55	5.81	37.07	1.14E-09	1.91E-07	liver
novel-ssc-miR-149-5p	-9.96	3.26	19.30	1.12E-05	9.94E-04	liver
novel-ssc-miR-168-3p	-7.63	5.00	19.60	9.55E-06	9.01E-04	liver
novel-ssc-miR-168-5p	-14.09	7.35	48.03	4.20E-12	1.59E-09	liver
novel-ssc-miR-178-5p	10.16	3.45	21.56	3.42E-06	3.98E-04	liver
novel-ssc-miR-190-3p	10.86	4.13	25.47	4.48E-07	5.64E-05	liver
novel-ssc-miR-234-3p	-8.86	2.21	14.07	1.77E-04	9.88E-03	liver
novel-ssc-miR-239-5p	-7.49	3.63	15.54	8.07E-05	5.08E-03	liver
novel-ssc-miR-36-3p	-7.21	5.05	18.46	1.73E-05	1.38E-03	liver
novel-ssc-miR-49-5p	-8.40	1.79	12.27	4.60E-04	2.17E-02	liver
novel-ssc-miR-58-3p	-11.72	4.99	29.42	5.82E-08	7.99E-06	liver
novel-ssc-miR-58-5p	-14.09	7.35	48.03	4.20E-12	1.59E-09	liver
novel-ssc-miR-64-5p	7.84	1.27	11.13	8.50E-04	3.57E-02	liver
novel-ssc-miR-81-5p	-9.83	3.14	18.62	1.60E-05	1.34E-03	liver
ssc-miR-206-5p	-8.49	1.87	12.62	3.82E-04	1.92E-02	liver
ssc-miR-22-3p	-2.16	15.22	5.38	1.41E-06	3.66E-04	liver
ssc-miR-4335-5p	-13.02	6.28	38.80	4.71E-10	9.29E-08	liver
conserve-ssc-miR-1246-3p	-7.90	0.65	10.80	1.02E-03	4.51E-02	heart
conserve-ssc-miR-1271-3p	-8.25	0.96	12.14	4.94E-04	2.87E-02	heart
conserve-ssc-miR-130a-5p	-13.41	5.99	47.10	6.73E-12	2.54E-09	heart
conserve-ssc-miR-191-3p	-8.51	1.20	13.24	2.75E-04	1.98E-02	heart
conserve-ssc-miR-2285ad-3p	-10.95	3.55	27.38	1.67E-07	3.03E-05	heart

conserve-ssc-miR-24-3-3p	-9.27	1.91	16.86	4.03E-05	3.81E-03	heart
conserve-ssc-miR-2902-5p	10.97	3.56	27.03	2.01E-07	3.03E-05	heart
conserve-ssc-miR-296-3p	-4.40	5.20	11.34	7.57E-04	3.68E-02	heart
conserve-ssc-miR-300-5p	7.98	0.72	10.88	9.73E-04	4.46E-02	heart
conserve-ssc-miR-378-3p	-8.13	0.85	11.67	6.35E-04	3.29E-02	heart
conserve-ssc-miR-4520-1-3p	12.26	4.84	36.92	1.23E-09	3.72E-07	heart
conserve-ssc-miR-4520-1-5p	9.06	1.72	15.46	8.43E-05	7.07E-03	heart
conserve-ssc-miR-500-5p	-9.99	2.61	21.30	3.93E-06	5.01E-04	heart
conserve-ssc-miR-541-1-5p	-8.79	1.46	14.46	1.43E-04	1.14E-02	heart
conserve-ssc-miR-6392-3p	-8.68	1.35	13.95	1.88E-04	1.42E-02	heart
conserve-ssc-miR-9098-1-3p	8.19	0.91	11.68	6.33E-04	3.29E-02	heart
conserve-ssc-miR-93-3p	-5.38	6.13	16.91	3.92E-05	3.81E-03	heart
conserve-ssc-miR-93-5p	-11.71	4.29	32.85	9.94E-09	2.15E-06	heart
novel-ssc-miR-124-3p	9.40	2.04	17.31	3.18E-05	3.43E-03	heart
novel-ssc-miR-144-3p	-9.43	2.07	17.81	2.44E-05	2.84E-03	heart
novel-ssc-miR-164-3p	-8.03	0.76	11.29	7.80E-04	3.68E-02	heart
novel-ssc-miR-195-5p	-8.45	1.14	12.96	3.18E-04	2.09E-02	heart
novel-ssc-miR-209-3p	9.11	1.77	15.74	7.28E-05	6.47E-03	heart
novel-ssc-miR-242-3p	8.32	1.03	12.18	4.84E-04	2.87E-02	heart
novel-ssc-miR-3-3p	11.75	9.23	63.68	1.46E-15	1.10E-12	heart
novel-ssc-miR-3-5p	13.88	6.46	50.81	1.02E-12	5.13E-10	heart
novel-ssc-miR-51-5p	-6.62	5.48	21.28	3.97E-06	5.01E-04	heart
ssc-miR-411-3p	-10.92	3.52	27.15	1.88E-07	3.03E-05	heart
ssc-miR-411-5p	-15.39	7.96	67.44	2.17E-16	3.28E-13	heart
ssc-miR-4331-5p	-8.44	1.14	12.96	3.19E-04	2.09E-02	heart
ssc-miR-503-5p	-8.12	0.84	11.62	6.54E-04	3.29E-02	heart
ssc-miR-551a-3p	-8.16	0.88	11.78	5.98E-04	3.29E-02	heart
ssc-miR-664-5p	12.20	4.79	36.44	1.58E-09	3.97E-07	heart
ssc-miR-7136-5p	4.49	5.96	12.62	3.82E-04	2.40E-02	heart
conserve-ssc-miR-101b-3p	-8.51	1.07	15.84	6.89E-05	4.53E-03	spleen
conserve-ssc-miR-1228-3p	6.51	2.66	10.85	9.86E-04	4.58E-02	spleen
conserve-ssc-miR-215-3p	-7.72	0.37	12.23	4.71E-04	2.54E-02	spleen
conserve-ssc-miR-2384-3p	-11.12	3.56	30.87	2.76E-08	5.22E-06	spleen
conserve-ssc-miR-2411-5p	-11.07	3.51	30.46	3.41E-08	5.73E-06	spleen
conserve-ssc-miR-24-3p	-8.74	1.28	16.90	3.94E-05	3.31E-03	spleen
conserve-ssc-miR-2456-3p	-13.51	5.92	60.51	7.34E-15	5.54E-12	spleen
conserve-ssc-miR-2456-5p	-12.59	5.00	46.25	1.04E-11	5.23E-09	spleen
conserve-ssc-miR-2902-3p	8.14	1.25	10.83	1.00E-03	4.58E-02	spleen
conserve-ssc-miR-378-5p	-11.20	3.63	31.47	2.03E-08	4.38E-06	spleen
conserve-ssc-miR-380-5p	7.36	3.53	14.38	1.49E-04	9.01E-03	spleen
conserve-ssc-miR-451b-3p	-7.85	0.49	12.83	3.41E-04	1.98E-02	spleen
conserve-ssc-miR-4675-3p	10.01	3.04	17.51	2.87E-05	2.55E-03	spleen
conserve-ssc-miR-6758-5p	9.44	2.48	15.25	9.44E-05	5.94E-03	spleen
conserve-ssc-miR-9173-3p	-10.05	2.51	23.66	1.15E-06	1.34E-04	spleen

conserve-ssc-miR-9307-3p	10.84	3.86	21.82	3.00E-06	3.02E-04	spleen
novel-ssc-miR-124-3p	11.34	4.35	24.94	5.91E-07	8.11E-05	spleen
novel-ssc-miR-133-5p	8.22	1.33	11.10	8.64E-04	4.21E-02	spleen
novel-ssc-miR-134-5p	-7.48	0.18	11.24	7.99E-04	4.03E-02	spleen
novel-ssc-miR-135-3p	13.07	6.07	41.18	1.39E-10	5.25E-08	spleen
novel-ssc-miR-176-3p	6.96	4.54	16.49	4.89E-05	3.69E-03	spleen
novel-ssc-miR-190-3p	9.65	2.69	16.07	6.10E-05	4.19E-03	spleen
novel-ssc-miR-36-3p	-8.59	1.14	16.20	5.69E-05	4.09E-03	spleen
novel-ssc-miR-47-3p	-10.13	2.59	24.17	8.83E-07	1.11E-04	spleen
novel-ssc-miR-70-5p	-7.00	2.61	16.66	4.47E-05	3.55E-03	spleen
novel-ssc-miR-74-5p	-8.88	1.41	17.58	2.76E-05	2.55E-03	spleen
novel-ssc-miR-94-3p	10.99	4.01	22.73	1.87E-06	2.01E-04	spleen
novel-ssc-miR-94-5p	5.15	5.07	12.37	4.36E-04	2.44E-02	spleen
ssc-miR-1468-5p	-2.23	10.26	11.26	7.91E-04	4.03E-02	spleen
ssc-miR-411-3p	-10.67	3.12	27.47	1.59E-07	2.41E-05	spleen
ssc-miR-411-5p	-15.68	8.09	116.81	3.15E-27	4.76E-24	spleen
ssc-miR-4335-5p	-11.89	4.31	37.90	7.46E-10	2.25E-07	spleen
ssc-miR-497-5p	12.46	5.46	34.52	4.23E-09	1.06E-06	spleen
conserve-ssc-miR-2285ad-3p	12.99	5.62	72.40	1.76E-17	1.33E-14	lung
conserve-ssc-miR-2902-3p	-8.88	1.60	19.15	1.21E-05	9.14E-04	lung
conserve-ssc-miR-500-5p	-9.16	1.86	21.10	4.37E-06	4.71E-04	lung
conserve-ssc-miR-5121-3p	7.47	0.31	12.42	4.24E-04	1.83E-02	lung
conserve-ssc-miR-532-1-5p	8.14	0.92	15.74	7.26E-05	4.22E-03	lung
conserve-ssc-miR-6322-3p	-7.84	0.65	13.86	1.97E-04	9.31E-03	lung
conserve-ssc-miR-644a-5p	-9.80	2.48	26.52	2.61E-07	3.29E-05	lung
conserve-ssc-miR-8884-5p	-7.47	0.32	12.06	5.14E-04	2.10E-02	lung
conserve-ssc-miR-9104-3p	-8.72	1.45	18.26	1.93E-05	1.33E-03	lung
conserve-ssc-miR-9173-5p	6.37	1.66	14.03	1.79E-04	8.75E-03	lung
conserve-ssc-miR-9b-5p	-7.56	0.41	12.54	3.98E-04	1.77E-02	lung
novel-ssc-miR-12-5p	7.92	0.71	14.64	1.30E-04	6.56E-03	lung
novel-ssc-miR-161-3p	-9.80	2.48	26.52	2.61E-07	3.29E-05	lung
novel-ssc-miR-164-3p	-9.25	1.95	21.80	3.03E-06	3.53E-04	lung
novel-ssc-miR-168-5p	-8.41	1.16	16.61	4.59E-05	2.77E-03	lung
novel-ssc-miR-172-5p	-7.20	0.11	10.80	1.01E-03	4.03E-02	lung
novel-ssc-miR-180-5p	8.93	1.64	19.99	7.78E-06	6.53E-04	lung
novel-ssc-miR-19-5p	7.41	0.26	12.11	5.01E-04	2.10E-02	lung
novel-ssc-miR-198-5p	8.11	0.88	15.56	8.00E-05	4.32E-03	lung
novel-ssc-miR-199-3p	-8.96	1.68	19.65	9.30E-06	7.40E-04	lung
novel-ssc-miR-199-5p	-7.58	0.42	12.63	3.80E-04	1.74E-02	lung
novel-ssc-miR-209-5p	8.93	1.64	19.99	7.78E-06	6.53E-04	lung
novel-ssc-miR-232-3p	9.93	2.60	28.43	9.71E-08	1.63E-05	lung
novel-ssc-miR-234-5p	-8.56	1.30	17.41	3.01E-05	1.98E-03	lung
novel-ssc-miR-239-5p	8.93	1.64	19.99	7.78E-06	6.53E-04	lung
novel-ssc-miR-247-3p	9.93	2.60	28.43	9.71E-08	1.63E-05	lung

novel-ssc-miR-33-5p	-9.10	1.80	20.63	5.58E-06	5.62E-04	lung
novel-ssc-miR-51-3p	-8.04	0.82	14.75	1.23E-04	6.39E-03	lung
novel-ssc-miR-58-5p	-8.41	1.16	16.61	4.59E-05	2.77E-03	lung
novel-ssc-miR-70-5p	-9.80	2.48	26.52	2.61E-07	3.29E-05	lung
novel-ssc-miR-82-3p	-6.62	2.46	18.36	1.83E-05	1.31E-03	lung
novel-ssc-miR-82-5p	8.12	0.90	15.62	7.75E-05	4.32E-03	lung
ssc-miR-144-3p	-13.75	6.38	82.09	1.30E-19	1.97E-16	lung
ssc-miR-144-5p	-12.94	5.57	70.41	4.82E-17	1.82E-14	lung
ssc-miR-331-3p	11.01	3.66	40.47	2.00E-10	6.03E-08	lung
ssc-miR-331-5p	10.71	3.36	36.82	1.30E-09	3.26E-07	lung
ssc-miR-411-5p	-13.03	5.66	71.55	2.70E-17	1.36E-14	lung
ssc-miR-4335-5p	-10.70	3.35	35.79	2.20E-09	4.75E-07	lung
conserve-ssc-miR-1777b-5p	9.86	2.58	19.78	8.68E-06	5.05E-04	duodenum
conserve-ssc-miR-181b-3p	7.94	0.77	11.01	9.08E-04	2.69E-02	duodenum
conserve-ssc-miR-216b-3p	-7.59	5.44	26.16	3.15E-07	3.66E-05	duodenum
conserve-ssc-miR-216b-5p	-7.35	6.55	29.80	4.80E-08	9.06E-06	duodenum
conserve-ssc-miR-2285ad-3p	-8.50	1.30	11.87	5.70E-04	1.83E-02	duodenum
conserve-ssc-miR-2411-1-5p	9.86	2.58	19.78	8.68E-06	5.05E-04	duodenum
conserve-ssc-miR-2456-3p	11.19	3.88	29.07	7.00E-08	1.06E-05	duodenum
conserve-ssc-miR-2456-5p	11.47	4.15	31.35	2.15E-08	4.65E-06	duodenum
conserve-ssc-miR-24-5p	8.26	1.06	12.16	4.89E-04	1.61E-02	duodenum
conserve-ssc-miR-296-3p	6.42	2.07	10.58	1.14E-03	3.25E-02	duodenum
conserve-ssc-miR-299-3p	10.38	3.08	23.14	1.50E-06	1.26E-04	duodenum
conserve-ssc-miR-300-3p	-9.97	2.68	18.72	1.51E-05	7.61E-04	duodenum
conserve-ssc-miR-339a-3p	-9.14	1.89	14.39	1.49E-04	6.07E-03	duodenum
conserve-ssc-miR-3611-3p	-8.35	1.16	11.31	7.70E-04	2.33E-02	duodenum
conserve-ssc-miR-3674-5p	-7.92	0.78	9.85	1.70E-03	4.28E-02	duodenum
conserve-ssc-miR-4520-1-3p	-11.35	4.04	28.13	1.14E-07	1.43E-05	duodenum
conserve-ssc-miR-4520-1-5p	-9.01	1.77	13.85	1.98E-04	7.88E-03	duodenum
conserve-ssc-miR-4722-5p	9.13	1.87	15.77	7.16E-05	3.18E-03	duodenum
conserve-ssc-miR-504-3p	8.48	1.26	13.00	3.11E-04	1.20E-02	duodenum
conserve-ssc-miR-532-1-5p	8.29	1.09	12.28	4.59E-04	1.54E-02	duodenum
conserve-ssc-miR-574-3p	-7.96	0.81	9.97	1.59E-03	4.14E-02	duodenum
conserve-ssc-miR-6322-5p	8.29	1.09	12.28	4.59E-04	1.54E-02	duodenum
conserve-ssc-miR-6525-3p	12.65	5.32	43.28	4.74E-11	3.58E-08	duodenum
conserve-ssc-miR-8884-3p	-8.35	1.16	11.31	7.70E-04	2.33E-02	duodenum
conserve-ssc-miR-8915-3p	7.78	0.62	10.43	1.24E-03	3.40E-02	duodenum
conserve-ssc-miR-9173-3p	7.63	4.94	24.08	9.22E-07	8.20E-05	duodenum
conserve-ssc-miR-9173-5p	10.23	2.93	22.11	2.57E-06	1.85E-04	duodenum
novel-ssc-miR-112-5p	-7.97	0.82	10.01	1.56E-03	4.13E-02	duodenum
novel-ssc-miR-121-3p	9.77	2.48	19.20	1.18E-05	6.35E-04	duodenum
novel-ssc-miR-135-3p	-10.47	3.18	21.90	2.87E-06	1.97E-04	duodenum
novel-ssc-miR-154-3p	-9.45	2.19	15.90	6.67E-05	3.05E-03	duodenum
novel-ssc-miR-163-3p	9.09	1.83	15.58	7.92E-05	3.42E-03	duodenum

novel-ssc-miR-173-3p	9.75	2.46	19.08	1.26E-05	6.55E-04	duodenum
novel-ssc-miR-180-3p	12.28	4.96	39.15	3.92E-10	1.98E-07	duodenum
novel-ssc-miR-180-5p	10.23	2.93	22.11	2.57E-06	1.85E-04	duodenum
novel-ssc-miR-181-5p	7.88	0.72	10.80	1.02E-03	2.96E-02	duodenum
novel-ssc-miR-212-5p	9.86	2.58	19.78	8.68E-06	5.05E-04	duodenum
novel-ssc-miR-217-3p	9.40	2.13	17.10	3.54E-05	1.67E-03	duodenum
novel-ssc-miR-232-3p	16.32	8.99	88.02	6.48E-21	9.80E-18	duodenum
novel-ssc-miR-232-5p	9.00	1.75	15.20	9.65E-05	4.05E-03	duodenum
novel-ssc-miR-234-5p	8.39	1.18	12.68	3.71E-04	1.37E-02	duodenum
novel-ssc-miR-237-3p	7.63	4.94	24.08	9.22E-07	8.20E-05	duodenum
novel-ssc-miR-237-5p	10.23	2.93	22.11	2.57E-06	1.85E-04	duodenum
novel-ssc-miR-239-3p	7.63	4.94	24.08	9.22E-07	8.20E-05	duodenum
novel-ssc-miR-239-5p	11.15	3.83	28.71	8.39E-08	1.15E-05	duodenum
novel-ssc-miR-36-3p	-8.35	1.16	11.31	7.70E-04	2.33E-02	duodenum
novel-ssc-miR-39-5p	8.38	1.17	12.62	3.82E-04	1.37E-02	duodenum
novel-ssc-miR-43-3p	9.95	2.67	20.34	6.49E-06	4.26E-04	duodenum
novel-ssc-miR-51-5p	7.53	5.18	24.90	6.04E-07	6.52E-05	duodenum
novel-ssc-miR-5-3p	-10.05	2.77	19.25	1.15E-05	6.35E-04	duodenum
novel-ssc-miR-56-5p	12.13	4.81	37.56	8.86E-10	3.35E-07	duodenum
novel-ssc-miR-77-3p	8.41	1.19	12.73	3.60E-04	1.36E-02	duodenum
ssc-miR-129a-5p	-9.84	2.56	17.97	2.25E-05	1.10E-03	duodenum
ssc-miR-144-3p	-8.64	1.42	12.38	4.33E-04	1.52E-02	duodenum
ssc-miR-216-3p	-11.53	4.21	29.57	5.40E-08	9.06E-06	duodenum
ssc-miR-216-5p	-8.84	5.60	32.42	1.24E-08	3.75E-06	duodenum
ssc-miR-217-3p	-8.01	0.85	10.14	1.45E-03	3.91E-02	duodenum
ssc-miR-217-5p	-11.80	4.48	31.93	1.60E-08	4.03E-06	duodenum
ssc-miR-331-3p	-8.12	0.95	10.51	1.19E-03	3.32E-02	duodenum
ssc-miR-331-5p	-7.92	0.78	9.85	1.70E-03	4.28E-02	duodenum
conserve-ssc-miR-106a-2-3p	-9.45	2.46	22.76	1.84E-06	2.53E-04	*longissimus dorsi*
conserve-ssc-miR-106a-2-5p	-13.60	6.54	49.58	1.91E-12	1.44E-09	*longissimus dorsi*
conserve-ssc-miR-1228-3p	5.32	6.59	17.68	2.61E-05	2.85E-03	*longissimus dorsi*
conserve-ssc-miR-130a-5p	11.57	4.52	38.07	6.83E-10	1.29E-07	*longissimus dorsi*
conserve-ssc-miR-2331-5p	-8.08	1.19	13.41	2.50E-04	2.10E-02	*longissimus dorsi*
conserve-ssc-miR-2893-3p	11.95	4.90	40.29	2.19E-10	5.51E-08	*longissimus dorsi*
conserve-ssc-miR-2893-5p	12.82	5.76	45.77	1.33E-11	6.71E-09	*longissimus dorsi*
conserve-ssc-miR-500-5p	-12.11	5.07	39.35	3.54E-10	7.64E-08	*longissimus dorsi*

conserve-ssc-miR-6527-3p	7.80	0.92	12.67	3.71E-04	2.80E-02	*longissimus dorsi*
conserve-ssc-miR-6758-5p	8.04	1.14	14.11	1.72E-04	1.53E-02	*longissimus dorsi*
conserve-ssc-miR-9100-5p	8.57	1.62	17.66	2.64E-05	2.85E-03	*longissimus dorsi*
novel-ssc-miR-176-3p	15.81	8.75	71.14	3.33E-17	5.04E-14	*longissimus dorsi*
novel-ssc-miR-47-3p	9.78	2.77	26.91	2.13E-07	3.21E-05	*longissimus dorsi*
novel-ssc-miR-5-3p	-5.10	3.82	14.32	1.54E-04	1.45E-02	*longissimus dorsi*
ssc-miR-1-3p	-3.43	12.41	12.02	5.25E-04	3.78E-02	*longissimus dorsi*
ssc-miR-206-5p	-3.59	12.22	12.91	3.27E-04	2.60E-02	*longissimus dorsi*
ssc-miR-4331-5p	8.73	1.77	18.88	1.39E-05	1.75E-03	*longissimus dorsi*
ssc-miR-4335-5p	12.36	5.31	42.70	6.37E-11	2.41E-08	*longissimus dorsi*
ssc-miR-503-3p	-8.62	1.68	16.88	3.97E-05	4.00E-03	*longissimus dorsi*
ssc-miR-503-5p	-12.54	5.49	41.87	9.74E-11	2.94E-08	*longissimus dorsi*
ssc-miR-664-5p	-10.39	3.37	29.71	5.03E-08	8.44E-06	*longissimus dorsi*

Supplementary Table S2. Tissue-specific DEmiRNAs in different tissues.

Tissue	miRNAs
Lung	conserve-ssc-miR-644a-5p
Lung	conserve-ssc-miR-6322-3p
Lung	conserve-ssc-miR-9b-5p
Heart	conserve-ssc-miR-2902-5p
Heart	conserve-ssc-miR-24-3-3p
Heart	conserve-ssc-miR-6392-3p
Heart	conserve-ssc-miR-191-3p
Heart	ssc-miR-7136-5p
Heart	conserve-ssc-miR-1271-3p
Heart	conserve-ssc-miR-9098-1-3p
Heart	conserve-ssc-miR-300-5p
Spleen	ssc-miR-497-5p
Spleen	conserve-ssc-miR-2411-5p
Spleen	conserve-ssc-miR-9307-3p
Spleen	conserve-ssc-miR-6758-5p
Liver	conserve-ssc-miR-4520-1-3p
Liver	conserve-ssc-miR-4520-1-5p
Liver	conserve-ssc-miR-8884-3p
Liver	conserve-ssc-miR-129-3p
Liver	conserve-ssc-miR-4303-3p
Liver	conserve-ssc-miR-1933-2-5p
Liver	conserve-ssc-miR-646-3p
Liver	conserve-ssc-miR-18a-3p
Liver	conserve-ssc-miR-146a-1-5p
Liver	conserve-ssc-miR-1422o-3p
Duodenum	conserve-ssc-miR-6525-3p
Duodenum	ssc-miR-216-5p
Duodenum	ssc-miR-217-5p
Duodenum	conserve-ssc-miR-216b-5p
Duodenum	ssc-miR-216-3p
Duodenum	conserve-ssc-miR-216b-3p
Duodenum	conserve-ssc-miR-9173-3p
Duodenum	conserve-ssc-miR-299-3p
Duodenum	conserve-ssc-miR-9173-5p
Duodenum	conserve-ssc-miR-1777b-5p
Duodenum	conserve-ssc-miR-2411-1-5p
Duodenum	conserve-ssc-miR-4722-5p
Duodenum	conserve-ssc-miR-504-3p
Duodenum	conserve-ssc-miR-6322-5p
Duodenum	conserve-ssc-miR-24-5p
Duodenum	conserve-ssc-miR-3611-3p
Duodenum	conserve-ssc-miR-181b-3p

Duodenum	conserve-ssc-miR-8915-3p
Duodenum	ssc-miR-217-3p
Duodenum	conserve-ssc-miR-3674-5p
Longissimus dorsi	conserve-ssc-miR-106a-2-5p
Longissimus dorsi	conserve-ssc-miR-2893-5p
Longissimus dorsi	ssc-miR-503-5p
Longissimus dorsi	conserve-ssc-miR-2893-3p
Longissimus dorsi	conserve-ssc-miR-500-5p
Longissimus dorsi	conserve-ssc-miR-106a-2-3p
Longissimus dorsi	conserve-ssc-miR-1228-3p
Longissimus dorsi	conserve-ssc-miR-9100-5p
Longissimus dorsi	ssc-miR-503-3p
Longissimus dorsi	conserve-ssc-miR-2331-5p
Longissimus dorsi	ssc-miR-206-5p
Longissimus dorsi	conserve-ssc-miR-6527-3p
Longissimus dorsi	ssc-miR-1-3p

Supplementary Table S3. Functional enrichment of genes targeted by tissue-specific miRNAs which differentially expressed with no overlap among tissues

Tissue	Category	Term	Count	*P* value
heart	GO-BP	heart development	127	0.000943
heart	GO-BP	heart looping	47	0.002686
heart	GO-BP	ventricular septum development	22	0.042874
heart	KEGG	Adrenergic signaling in cardiomyocytes	95	0.01084
heart	GO-BP	fibroblast growth factor receptor signaling pathway	57	0.028753
liver	KEGG	Insulin resistance	90	3.58E-07
liver	KEGG	Insulin signaling pathway	106	3.95E-05
liver	GO-BP	glucose metabolic process	50	0.012311
liver	GO-BP	gluconeogenesis	33	0.044386
spleen	KEGG	T cell receptor signaling pathway	84	1.37E-08
spleen	KEGG	B cell receptor signaling pathway	54	0.00034
spleen	GO-BP	immunoglobulin mediated immune response	11	0.015761
lung	GO-BP	angiogenesis	163	9.76E-07
lung	GO-BP	lung development	60	0.000145
lung	GO-BP	lung alveolus development	26	0.036624
duodenum	GO-BP	positive regulation of epithelial cell proliferation	50	0.000607
duodenum	KEGG	Bacterial invasion of epithelial cells	65	0.000097
longissimus dorsi	GO-BP	skeletal muscle cell differentiation	39	0.002239
longissimus dorsi	GO-BP	muscle fiber development	12	0.037281

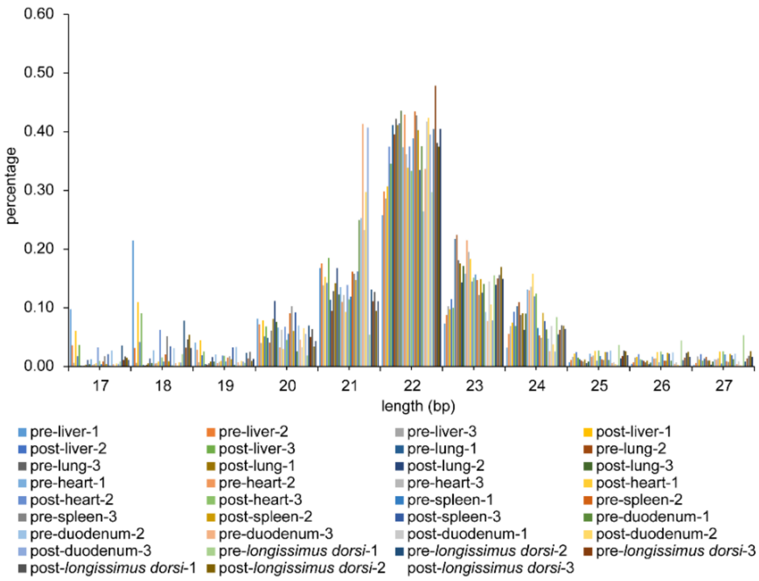

Supplementary Figure S1 Length distribution of sequenced high quality reads for each sample.

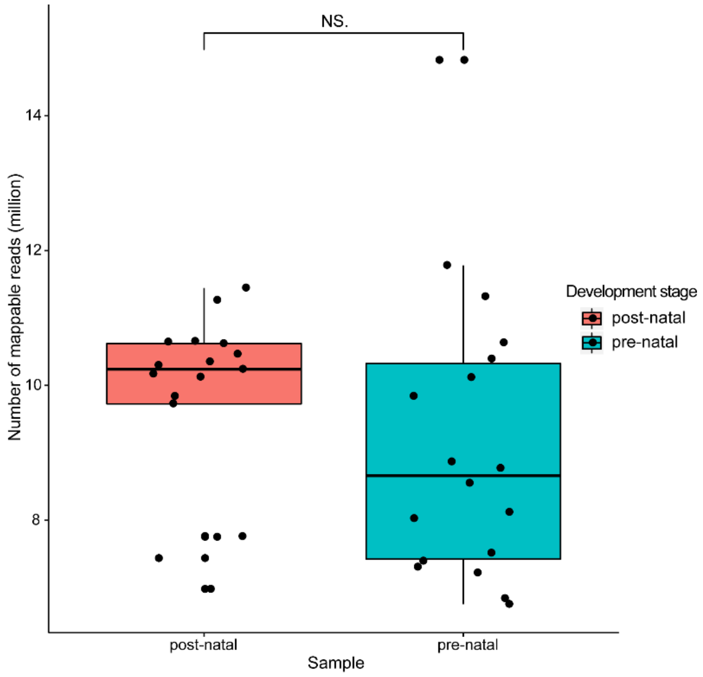

Supplementary Figure S2 Number of mappable reads of samples from pre- and post-natal piglets populations.

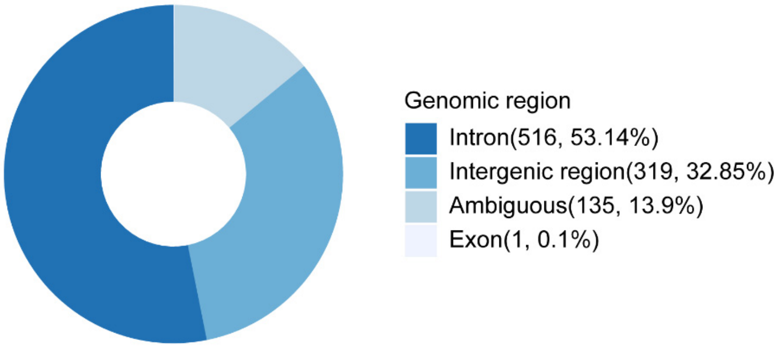

Supplementary Figure S3 Genomic sources of miRNA precursor sequences. 'Ambiguous': miRNA precursor sequences that locate in both introns and exons due to alternative splicing of protein-coding genes.

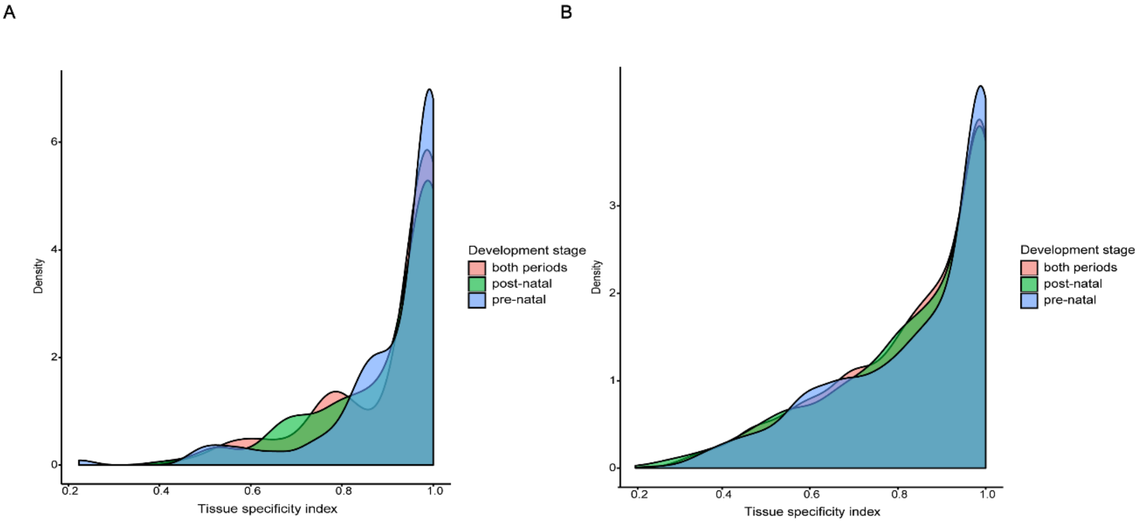

Supplementary Figure S4 Distribution curve for the frequency of TSI of (A) DE miRNAs and (B) non-DE miRNAs.

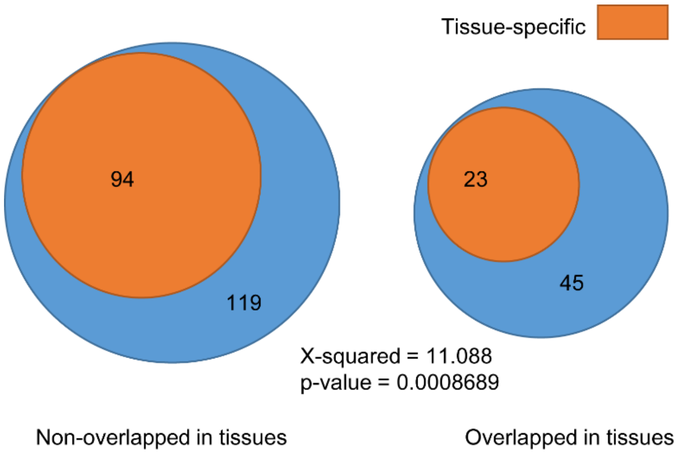

Supplementary Figure S5 Number of DE miRNAs with tissue-specificity and overlap in tissues. The P value was calculated using the Pearson's Chi-squared test with Yates' continuity correction.
